# Chronic hypoxia impairs skeletal muscle repair via HIF‐2α stabilization

**DOI:** 10.1002/jcsm.13436

**Published:** 2024-02-09

**Authors:** Amelia Yin, Wenyan Fu, Anthony Elengickal, Joonhee Kim, Yang Liu, Anne Bigot, Kamal Mamchaoui, Jarrod A. Call, Hang Yin

**Affiliations:** ^1^ Center for Molecular Medicine The University of Georgia Athens GA USA; ^2^ Department of Biochemistry and Molecular Biology The University of Georgia Athens GA USA; ^3^ Sorbonne Université, Inserm, Institut de Myologie Centre de Recherche en Myologie Paris France; ^4^ Department of Physiology and Pharmacology The University of Georgia Athens GA USA

**Keywords:** Angiotensin converting enzyme, Hypoxia, Hypoxia‐inducible factor 2A, Muscle atrophy, Muscle regeneration, Muscle stem cells

## Abstract

**Background:**

Chronic hypoxia and skeletal muscle atrophy commonly coexist in patients with COPD and CHF, yet the underlying physio‐pathological mechanisms remain elusive. Muscle regeneration, driven by muscle stem cells (MuSCs), holds therapeutic potential for mitigating muscle atrophy. This study endeavours to investigate the influence of chronic hypoxia on muscle regeneration, unravel key molecular mechanisms, and explore potential therapeutic interventions.

**Methods:**

Experimental mice were exposed to prolonged normobaric hypoxic air (15% *p*O_2_, 1 atm, 2 weeks) to establish a chronic hypoxia model. The impact of chronic hypoxia on body composition, muscle mass, muscle strength, and the expression levels of hypoxia‐inducible factors HIF‐1α and HIF‐2α in MuSC was examined. The influence of chronic hypoxia on muscle regeneration, MuSC proliferation, and the recovery of muscle mass and strength following cardiotoxin‐induced injury were assessed. The muscle regeneration capacities under chronic hypoxia were compared between wildtype mice, MuSC‐specific HIF‐2α knockout mice, and mice treated with HIF‐2α inhibitor PT2385, and angiotensin converting enzyme (ACE) inhibitor lisinopril. Transcriptomic analysis was performed to identify hypoxia‐ and HIF‐2α‐dependent molecular mechanisms. Statistical significance was determined using analysis of variance (ANOVA) and Mann–Whitney *U* tests.

**Results:**

Chronic hypoxia led to limb muscle atrophy (EDL: 17.7%, *P* < 0.001; Soleus: 11.5% reduction in weight, *P* < 0.001) and weakness (10.0% reduction in peak‐isometric torque, *P* < 0.001), along with impaired muscle regeneration characterized by diminished myofibre cross‐sectional areas, increased fibrosis (*P* < 0.001), and incomplete strength recovery (92.3% of pre‐injury levels, *P* < 0.05). HIF‐2α stabilization in MuSC under chronic hypoxia hindered MuSC proliferation (26.1% reduction of MuSC at 10 dpi, *P* < 0.01). HIF‐2α ablation in MuSC mitigated the adverse effects of chronic hypoxia on muscle regeneration and MuSC proliferation (30.9% increase in MuSC numbers at 10 dpi, *P* < 0.01), while HIF‐1α ablation did not have the same effect. HIF‐2α stabilization under chronic hypoxia led to elevated local ACE, a novel direct target of HIF‐2α. Notably, pharmacological interventions with PT2385 or lisinopril enhanced muscle regeneration under chronic hypoxia (PT2385: 81.3% increase, *P* < 0.001; lisinopril: 34.6% increase in MuSC numbers at 10 dpi, *P* < 0.05), suggesting their therapeutic potential for alleviating chronic hypoxia‐associated muscle atrophy.

**Conclusions:**

Chronic hypoxia detrimentally affects skeletal muscle regeneration by stabilizing HIF‐2α in MuSC and thereby diminishing MuSC proliferation. HIF‐2α increases local ACE levels in skeletal muscle, contributing to hypoxia‐induced regenerative deficits. Administration of HIF‐2α or ACE inhibitors may prove beneficial to ameliorate chronic hypoxia‐associated muscle atrophy and weakness by improving muscle regeneration under chronic hypoxia.

## Background

Localized and systemic hypoxia arise from environmental oxygen reduction, impaired gas exchange, compromised heart function, and localized circulatory defects. While transient hypoxia may be necessary for certain cellular functions, chronic hypoxia usually results in malfunctions and pathological adaptations, including oxidative stress, inflammation, and fibrosis.^1^ These are commonly observed in the skeletal muscle of patients with systemic chronic hypoxia such as chronic obstructive pulmonary disease (COPD)^2^ or chronic heart failure (CHF),^3^ as well as individuals with localized ischemia resulting from atherosclerosis, thrombosis, or haemorrhage.^4^

Hypoxia exerts profound effects on skeletal muscle.^5^ Exposure to hypoxic air is used in athletic training (S1), wherein hypoxia adaptations increase capacities in oxidative metabolism and oxygen transport, beneficial for muscle performance. However, prolonged hypoxia in COPD and CHF patients are associated with reduced exercise capacity, muscle weakness, and wasting (cachexia).^3^, ^6^ The divergence likely reflects fundamental differences in molecular mechanisms and cellular behaviours in responses to hypoxia at different oxygen tension levels and durations.

When considering the impacts of hypoxia on cachexia, the effects on supporting cell types in muscle other than contractile myofibres should not be overlooked.^7^ Skeletal muscle stem cells (MuSCs), also known as satellite cells, play a crucial role in repairing and regenerating skeletal muscle following traumatic and pathological conditions.^8^ With a low level of muscle repairing needs (e.g., resting and stationary lifestyle), MuSCs are mainly quiescent; upon myofibre damage (e.g., resistance training and injuries), MuSCs are activated and proliferate as myoblasts, which later undergo myogenic differentiation to repair damaged myonuclei/myofibres. Correspondingly, MuSCs may have a minimal role in maintaining the mass and strength of healthy muscles (S2–4). However, in conditions of diseases or aging, MuSCs play a critical role in myofibre repairing and regeneration (S5–6). Promising approaches to reverse cachexia/sarcopenia have demonstrated significant improvements in MuSCs function (S7–10), underscoring the vital therapeutic potential of MuSCs under disease conditions.

Accumulating evidence demonstrates that MuSCs dysfunction underlie hypoxia‐associated cachexia in COPD/CHF patients.^9^, ^10^ MuSCs isolated from COPD patients showed increased oxidative stress, defective autophagy, and senescence phenotypes,^11^, ^12^, ^13^ suggesting diminished regenerative potential. Causal evidence for hypoxia and MuSCs dysfunction is limited. By far, only one study reported that mice adapted to hypobaric hypoxia exhibited impaired muscle regeneration with a reduced number of proliferative myoblasts during regeneration.^14^

HIF‐1α and HIF‐2α are extensively studied isoforms of the hypoxia‐inducible factors (HIFα) family. Considerable evidence indicates that these two HIFα isoforms possess unique functions and are subject to distinct regulatory mechanisms (S11–12). Within myogenic cells, HIF‐1α has been shown to enhance myoblast proliferation while impeding myogenic differentiation, both *in vitro* (S13) and *in vivo* (S14–15). HIF‐1α is transiently expressed in activated/proliferative myoblasts within regenerative muscle (2–3 days post‐injury; dpi), when local hypoxia occurs^15^ (S16). In a later stage, HIF‐1α blocks the maturation of newly formed myofibres (S17). Conversely, our previous research revealed that HIF‐2α is expressed in quiescent MuSCs, which exist in an intracellular hypoxic state.^15^ Upon different types of muscle injuries, HIF‐2α expression diminishes to varying extents. Following muscle strains, HIF‐2α disappears in almost all MuSCs by 2 dpi, leading to synchronized MuSCs activation/proliferation. However, after cardiotoxin (CTX)‐induced damage, HIF‐2α expression is only reduced in a subset of proliferative myoblasts. Remarkably, inhibition or genetic ablation of HIF‐2α in CTX‐damaged muscles results in substantial improvements in myoblast proliferation and subsequent myogenic differentiation, eventually leading to accelerated muscle function recovery.^15^

Our previous study has demonstrated the crucial role of HIF‐2α in muscle regeneration, highlighting it as a potential therapeutic target for muscle injuries. In this study, we established a mouse model of systemic chronic hypoxia. We revealed that chronic hypoxia led to excessive stabilization of HIF‐2α in MuSCs, consequently impairing muscle regeneration. Intriguingly, targeted HIF‐2α ablation in MuSCs or transient antagonism by PT2385 rescued hypoxia‐associated regeneration defects and resulted in a remarkable restoration of muscle mass and strength. Mechanistically, HIF‐2α transactivates ACE in limb muscles under chronic hypoxia. ACE antagonism also showed beneficial effects on muscle regeneration under hypoxia, albeit to a lesser extent compared with PT2385. Our study provides valuable insights into the underlying causal relationships between chronic hypoxia and cachexia. Moreover, it elucidates the mechanistic basis for the observed clinical benefits of ACE inhibitors in COPD/CHF patients and provides proof of principle for a novel HIF‐2α antagonism‐based therapeutic strategy. These findings warrant further investigations into the adaptative mechanisms of skeletal muscle in response to chronic hypoxia and the corresponding therapeutic approaches.

## Methods

### Chronic hypoxia treatment

Mice subjected to chronic hypoxia treatment were individually housed in sealable metabolic chambers (L/W/H: 25 cm/15 cm/17 cm) with porous raised floors. The chambers received inlet gas from a two‐to‐one gas mixer/regulator, which was connected to a pressured air tank (20.9% *p*O_2_) and a nitrogen gas tank (100% N_2_). The *p*O_2_ in the inlet mixed air was adjusted by modulating the ratio between the two gas sources and continuously measured/monitored by an indirect calorimetry system (Oxymax, Columbus Instruments). The airflow to each metabolic chamber was set to 0.4 L/min.

For the first 5 days of the hypoxia adaptation stage, the *p*O_2_ in the inlet mixed air was gradually reduced: day_1 = 19%, day_2 = 17.5%, day_3 = 16.5%, day_4 = 15.5%, day_5 = 14.5–15%. Subsequently, the *p*O_2_ was maintained at 15 ± 0.3% (see Figure S1).

The mice in the ‘normoxia’ and ‘hypoxia’ groups had *ad libitum* access to chow. To replicate the hypoxia‐associated reduction of food intake under normoxia (‘normoxia+CR’ group), daily food consumption of mice under the above hypoxia treatment regime was monitored and recorded in pilot experiments. In subsequent experiments, mice in the ‘normoxia+CR’ group were provided with limited amounts of chow that matched to the recorded daily consumption under hypoxia.

### Statistics

Statistical analyses were conducted using Prism GraphPad 9.0. When specific pairs of groups are compared, Student's *t*‐tests were conducted to test the equality of the means following Shapiro–Wilk normality tests. When three or more groups are compared, one‐way ANOVA was employed to assess the significance of differences among multiple groups with significant level (α) set at 0.05. In case of significant differences in ANOVA, post hoc Tukey's HSD tests were performed to identify differences between specific two groups. The non‐parametric Mann–Whitney *U* test was employed to assess the significance of differences between two distributions of myofibre cross‐sectional areas, which deviate from a normal distribution.

## Results

### A chronic normobaric hypoxia model for investigating hypoxia effect on muscle stem cell‐mediated regeneration in atrophic muscles

Cardiac compensation following hypobaric hypoxia led to better oxygenation in limb muscles, distinct from the poorer muscle oxygenation under normobaric hypoxia.^16^ As such, a chronic normobaric hypoxia protocol was implemented in this study: during the first stage, mice were subjected to two‐week hypoxia adaptation, in which the ambient *p*O_2_ gradually decreased from 20.9% to 15%; during the second stage, mice experienced a four‐week hypoxia exposure, in which muscle regeneration was tested (Figure S1).

The choice of 15% *p*O_2_ was based on a human study investigating normobaric hypoxia and muscle wasting,^17^ wherein healthy individuals subjected to bed rest and 14.1% normobaric hypoxia for 21 days manifested limb muscle atrophy. In our preliminary study, 14.5–15% *p*O_2_ level resulted in low SpO_2_ (93.2 ± 1.9%), which mimics COPD patients.^18^

**Figure 1:**
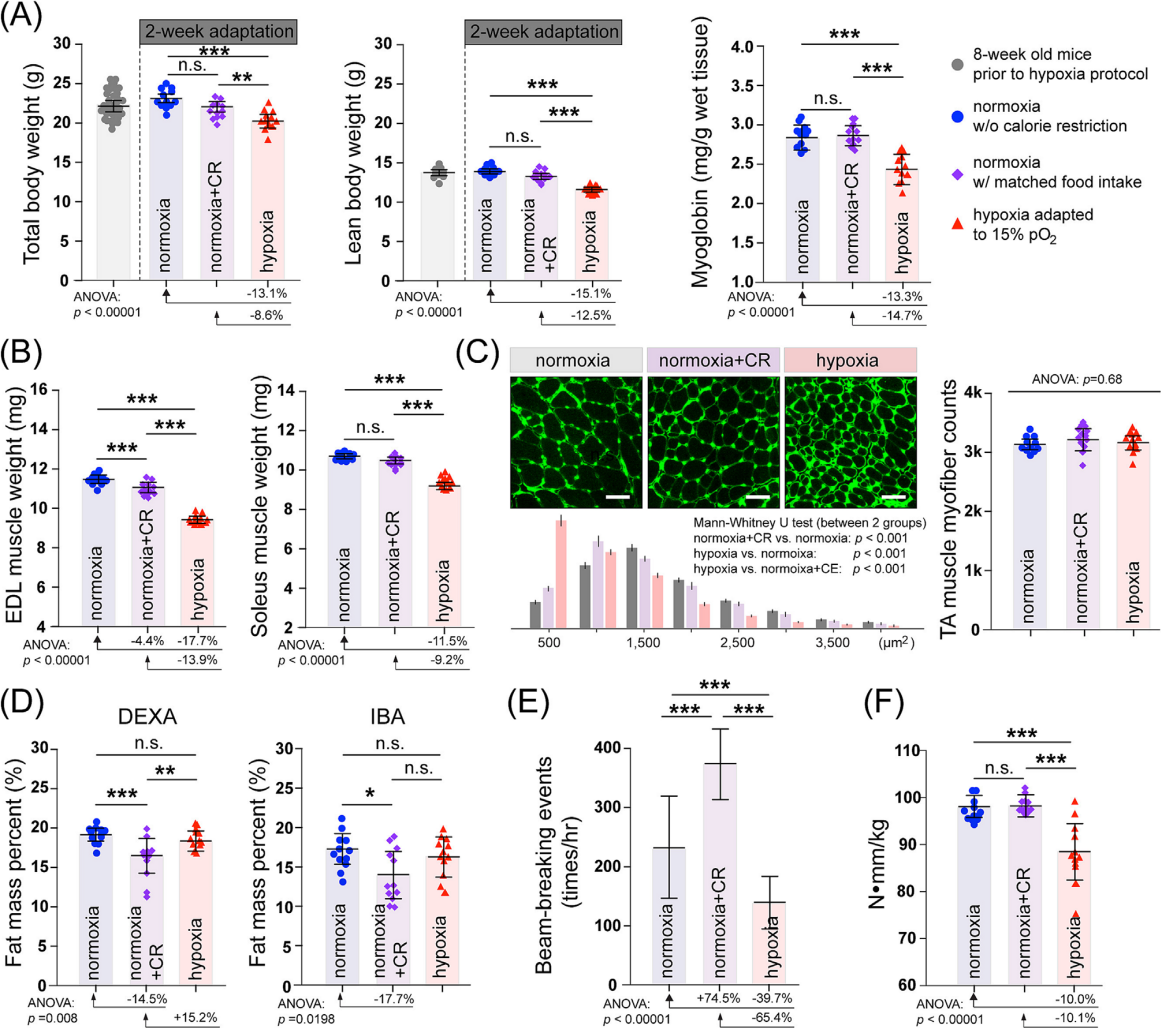
A chronic normobaric hypoxia model for investigating the effects of hypoxia on MuSCs‐mediated regeneration in atrophic muscles. (A) Left: Total body weight of *C57BL6/J* mice measured before (8‐week‐old; light grey; *n* = 36, 18 males and 18 females) and after a 2‐week chronic hypoxia adaptation stage (hypoxia, red; *n* = 12, 6 males and 6 females) or under normoxia with *ad libitum* food intake (normoxia, grey; *n* = 12, 6 males and 6 females) or matched food intake with the hypoxia group (normoxia+CR, purple; *n* = 12, 6 males and 6 females). Middle: Total lean body weight of the above‐described groups at the end of the 2‐week hypoxia adaptation stage (*n* = 12, 6 males and 6 females). Right: Myoglobin levels in TA muscle homogenates from the above‐described groups (*n* = 12, 6 males and 6 females). (B) Weight of EDL (left) and soleus (right) muscles of the three groups described in the panel (A) (*n* = 12, 6 males and 6 females). (C) Left: Upper panels show WGA staining on TA muscle cross‐sections of the three groups described in panel (A). Scale bar: 50 μm. The lower panel represents the distribution of myofibre cross‐sectional areas. Right: Quantification of myofibre numbers in the TA muscles. (*n* = 12, 6 males and 6 females) (D) fat mass percentages of mice in the three groups described in the panel (A) were measured using DEXA (left) or IBA (right) (*n* = 12, 6 males and 6 females). (E) Assessment of physical activities of mice in the three groups described in the panel (A) on the last day of the 2‐week chronic hypoxia adaptation stage. Histograms represent geometric means of beam‐breaking counts of the x‐, y‐, and z‐axis per hour (averaged for 24 data points per mouse) (*n* = 12, 6 males and 6 females). (F) Peak‐isometric torques of the TA/EDL muscle group of the three mice groups described in the panel (A) (*n* = 12, 6 males and 6 females). Statistical analysis legend: In panels (A), (B), (C, right), (D), (E), and (F), one‐way ANOVA was conducted to assess the equality of means among normoxia, normoxia+CR, and hypoxia groups, representing the null hypothesis. When *P*‐value < 0.05 (rejection of the null hypothesis), post hoc Tukey HSD tests were performed to determine significant differences between pairs of groups. The results of these post hoc tests have been denoted between the respective pairs of groups: ***P‐value < 0.001, **P‐value < 0.01, *P‐value < 0.05, n.s., not significant. Error bars represent standard deviations (SD). The percentages below histograms indicate the percent change (increase: +, decrease: ‐) compared to the condition depicted by the arrowheads, where statistically significant differences exist. In the panel (C, left lower), comparisons between normoxia vs. normoxia+CR groups, normoxia vs. hypoxia groups, and normoxia+CR vs. hypoxia groups were separately analysed by Mann–Whitney *U* tests due to their non‐normal distribution.

At the end of the hypoxia adaptation stage, we observed significant reductions in total and lean body weight compared with the pair‐fed normoxia group (normoxia+CR; Figure 1A, left and middle). The myoglobin levels in tibialis anterior (TA) muscles of the chronic hypoxia group also exhibited a significant reduction compared with the normoxia+CR group (Figure 1A, right). Notably, reductions in limb muscle weights (EDL and soleus) surpassed the overall lean body weight reduction in the hypoxia group compared with the normoxia+CR group (EDL: −17.7% vs. −4.4; soleus: −11.5% vs. −2.6%; Figure 1B). Histological examination of TA muscles confirmed a decrease in myofibre cross‐sectional areas (CSA) in the hypoxia group compared with both the normoxia group with *ad libitum* food intake and the normoxia+CR group, with no significant change in total myofibre numbers (Figure 1C). These indicate that chronic exposure to normobaric hypoxia leads to limb muscle atrophy, likely due to lower O_2_ tension independent of its indirect effect on food intake.

COPD patients show disproportional reductions in lean mass.^19^ In this study, dual‐energy X‐ray absorptiometry (DEXA) and bioelectrical impedance analysis (BIA) showed that hypoxia did not significantly alter fat mass percent, in contrast to a significant decrease in fat mass percent in the normoxia+CR group (DEXA: −14.5%, IBA: −17.7%; Figure 1D).

COPD patients typically exhibit reduced physical activity.^20^ The ambulatory activities were significantly lower in the hypoxia group compared with the normoxia+CR group (−65.4%; Figure 1E).

Muscle weakness is a common symptom in COPD patients with muscle cachexia. We measured the peak‐isometric torque generated by the TA/EDL muscle group after hypoxia adaptation. The maximal torque in the chronic hypoxia group was significantly lower than both normoxia control groups (−10.1 to 10.0%; Figure 1F).

The above results indicate that the chronic hypoxia mouse model exhibits similar muscular and behavioural phenotypes to COPD patients.

### Chronic hypoxia exposure impairs the proliferation and myogenic differentiation potential of muscle stem cell via HIF‐2A stabilization

We next explored the impacts of chronic hypoxia on MuSCs activation/proliferation and HIFα stabilization. First, we investigated MuSCs activation using *ex vivo* cultured single myofibres isolated from chronic hypoxia models. It has been reported that 10% normobaric hypoxia reduced the muscle oxygenation to 2.3% (compared with 6.8% under normoxia).^21^ To continue the chronic hypoxia exposure *ex vivo*, we cultured myofibres isolated from chronic hypoxia models under 4% *p*O_2_.

**Figure 2:**
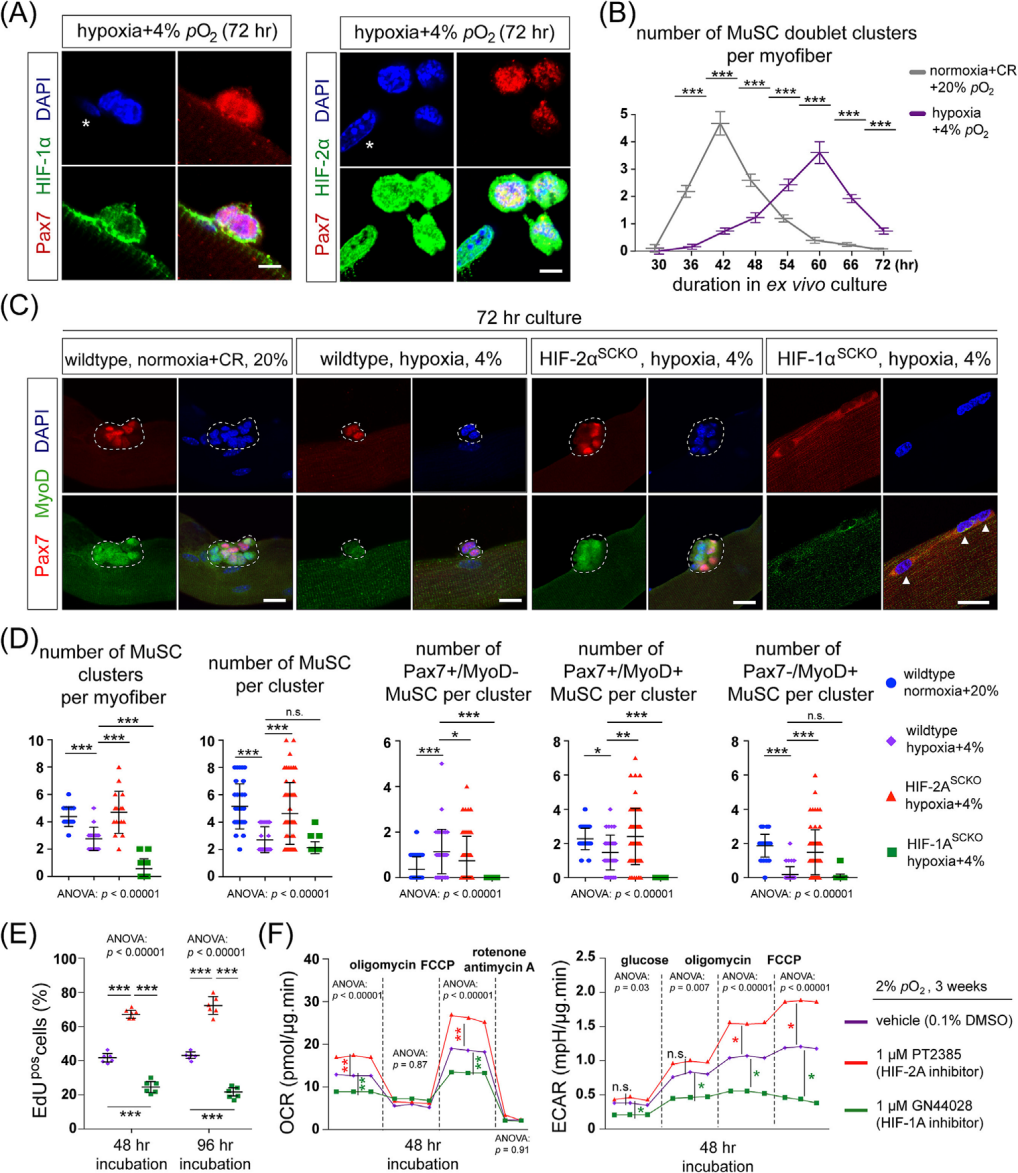
Chronic hypoxia exposure impairs the proliferation and myogenic differentiation potential of MuSCs via HIF‐2A stabilization. (A) Left: Immunostaining (IM) of Pax7 and HIF‐1α in a two‐cell (doublet) cluster of MuSCs on myofibres under chronic hypoxia. The myofibres were isolated from the chronic hypoxia models and cultured under 4% hypoxic air for 72 h. Asterisk: Pax7^neg^ myonucleus. Right: IM of Pax7 and HIF‐2α in a MuSCs cluster on myofibres under the same chronic hypoxia condition as the left panel. Asterisk: Pax7^neg^ myonucleus. Scale bar: 10 μm. Notably, myonuclei under this chronic hypoxia condition stained positively for HIF‐2α but not HIF‐1α. (B) Numbers of MuSCs doublet clusters on myofibres (*n* = 36–50) cultured under normoxia and hypoxia conditions during a 30–72‐hour post‐isolation window. For the normoxia condition, myofibres were isolated from mice in the normoxia+CR group (described in Figure 1) and cultured under 20% *p*O_2_. For the hypoxia condition, myofibres were isolated from the chronic hypoxia models (described in Figure 1) and cultured under 4% *p*O_2_. (C) IM of Pax7 and MyoD in representative MuSCs clusters under normoxic and hypoxic conditions. Wildtype: *C57BL6/J* mice. HIF‐1α^SCKO^: Tamoxifen‐induced *Pax7* ^*CreER*^ *; HIF‐1α* ^*flox/flox*^ mice. HIF‐2α^SCKO^: Tamoxifen‐induced *Pax7* ^*CreER*^ *; HIF‐2α* ^*flox/flox*^ mice. Arrowheads: Pax7^pos^ MuSCs. Scale bar: 20 μm. Notably, Pax7 and MyoD are present in the cytoplasm of MuSCs from HIF‐1α^SCKO^ myofibres exposed to chronic hypoxia. (D) From left to right: Numbers of MuSCs clusters per myofibre (*n* = 16) in the conditions depicted in panel (C). Numbers of MuSCs per cluster (*n* = 36–50) in the conditions depicted in panel (C). Numbers of Pax7^pos^/MyoD^neg^, Pax7^pos^/MyoD^pos^, and Pax7^neg^/MyoD^pos^ MuSCs in clusters (*n* = 36–50). (E) Percentages of EdU^pos^ human myoblasts, which were adapted to hypoxic culture (2% *p*O_2_ for 3 weeks), in 24‐h EdU pulse labelling experiments. Myoblasts were treated with either vehicle solution (0.1% DMSO; purple), 1 μM PT2385 (red), or 1 μM GN44028 (green) for 48 or 96 h under hypoxia (*n* = 6). (F) Left: A representative result of mitochondrial stress assays for hypoxia‐adapted human myoblasts under the same conditions as the panel (E) with 48 h of drug treatments (*n* = 7 per data point). Right: A representative result of extracellular acidification assays for hypoxia‐adapted human myoblasts under the same conditions as the panel (E) with 48 h of drug treatments (*n* = 7 per data point). Statistical analysis legend: In the panel (B) right, Student's *t*‐tests were conducted to assess the equality of means between the normoxia+CR and hypoxia groups. The results of *t*‐tests are indicated above the crossbars: ***P‐value < 0.001, **P‐value < 0.01, *P‐value < 0.05, n.s., not significant. In panels (D–F), one‐way ANOVA was conducted to assess the equality of means among groups. When ANOVA *P*‐value is less than 0.05, post hoc Tukey HSD tests were performed to determine significant differences between pairs of groups. The results of these post hoc tests have been denoted between the respective pairs of groups: ***P‐value < 0.001, **P‐value < 0.01, *P‐value < 0.05, n.s., not significant. Error bars represent standard deviations (SD).

Our previous research showed that HIF‐1α is not expressed in quiescent MuSCs.^15^ Under chronic hypoxia conditions, HIF‐1α was detected in activated MuSCs within clusters/doublets, although mostly localized in the cytoplasm (Figure 2A). In contrast, HIF‐2α was detected in both the cytosol and nucleus (Figure 2A). Compared with normoxic culture,^15^ HIF‐2α was notably enriched in the nucleus, suggesting its involvement in transcriptional regulation.

Chronic hypoxia negatively affected MuSCs activation/proliferation. The initial division of MuSCs on hypoxic myofibres was delayed to ~60 h after isolation compared with ~42 h under normoxia (Figure 2B). Additionally, MuSCs on myofibres underwent two to three divisions and formed four to eight cell clusters during 72 h‐normoxic culturing; however, MuSCs formed noticeably smaller clusters under hypoxia (Figure 2C).

As both HIF‐1α and HIF‐2α were stabilized, we aimed to determine which HIFα(s) contributed to the activation/proliferation defects. We utilized the chronic hypoxia protocol on Pax7^CreER^;HIF‐1α^flox/flox^ and Pax7^CreER^;HIF‐2α^flox/flox^ mice and induced HIFα (HIFα^SCKO^) knockout specifically in MuSCs during chronic hypoxia adaptation. Interestingly, HIF‐2α ablation rescued the activation/proliferation defects under hypoxia, as evidenced by the number and size of MuSCs clusters compared with the normoxic and hypoxic wildtype conditions; conversely, HIF‐1α ablation resulted in a reduction in MuSCs cluster numbers and size (Figure 2C,D). These findings highlight the distinct functions of HIF‐1α and HIF‐2α in MuSCs under chronic hypoxia.

Chronic hypoxia expanded the pool of Pax7^pos^/MyoD^neg^ MuSCs that exhibit a high tendency to self‐renewal and return to quiescence (Figure 2D). Conversely, chronic hypoxia reduced the pools of Pax7^pos^/MyoD^pos^ and Pax7^neg^/MyoD^pos^ MuSCs, which are associated with increased proliferation and myogenic differentiation, respectively (Figure 2D). In MuSCs exposed to chronic hypoxia, HIF‐2α ablation reduced self‐renewal potential and enhanced proliferation and myogenic differentiation potential, thereby partially normalizing the effects of chronic hypoxia (Figure 2D).

As MuSCs on HIF‐1α^SCKO^ myofibres failed to form clusters under chronic hypoxia, we examined Pax7 and MyoD expression in these MuSCs as singlets/doublets. Both Pax7 and MyoD were expressed in HIF‐1α^SCKO^ MuSCs yet noticeably only present in the cytoplasm (Figure 2C), suggesting that HIF‐1α ablation may impair their myogenic lineage progression under chronic hypoxia.

To confirm the above effects in human cells, we cultured human myoblasts (S18) under chronic hypoxia (2% *p*O_2_, 3 weeks) and investigated their responses to HIF‐1α or HIF‐2α inhibition: HIF‐1α inhibitor GN44028 (IC_50_ = 14 nM) or HIF‐2α inhibitor PT2385 (IC_50_ = <50 nM) demonstrated robust inhibitory effects on their respective targets without an off‐target effect on each other (S19). Interestingly, HIF‐1α and HIF‐2α inhibition resulted in opposite effects: PT2385 promoted human myoblast proliferation under chronic hypoxia, while GN44028 impaired it (Figure 2E).

To explore HIF‐α's functions in hypoxia metabolism, we treated chronic hypoxia‐exposed human myoblasts with PT2385, GN44028, or vehicle for 48 h. Subsequent measurements of OCR and ECAR revealed that PT2385 increased basal and FCCP‐stimulated respiration while minimally affecting uncoupled respiration (Figure 2F). In contrast, GN44028 reduced mitochondrial respiration rates (Figure 2F). Additionally, ECAR, a surrogate indicator of anaerobic glycolysis, was found to be augmented in response to PT2385 (Figure 2F), consistent with HIF‐2α's negative influence on the proliferation of these cells. Conversely, GN44028 led to a pronounced reduction in ECAR (Figure 2F), in line with a pivotal role of HIF‐1α in glycolysis.

### The impaired proliferation/differentiation of muscle stem cells and regeneration failure under chronic hypoxia can be rescued by either muscle stem cell‐specific HIF‐2A ablation or PT2385

We next aimed to understand the effects of chronic hypoxia and HIF‐2α on muscle regeneration. To this end, we treated chronic hypoxia models with CTX, a toxin causes extensive muscle damage. This choice facilitates comparisons with our previous results from HIF‐2α antagonism under normoxia.^15^ After one CTX injection to the TA muscles, the hypoxia models were continuously housed under normobaric hypoxia (15% *p*O_2_) for a maximum of 4 weeks until the endpoints (Figure S1).

**Figure 3:**
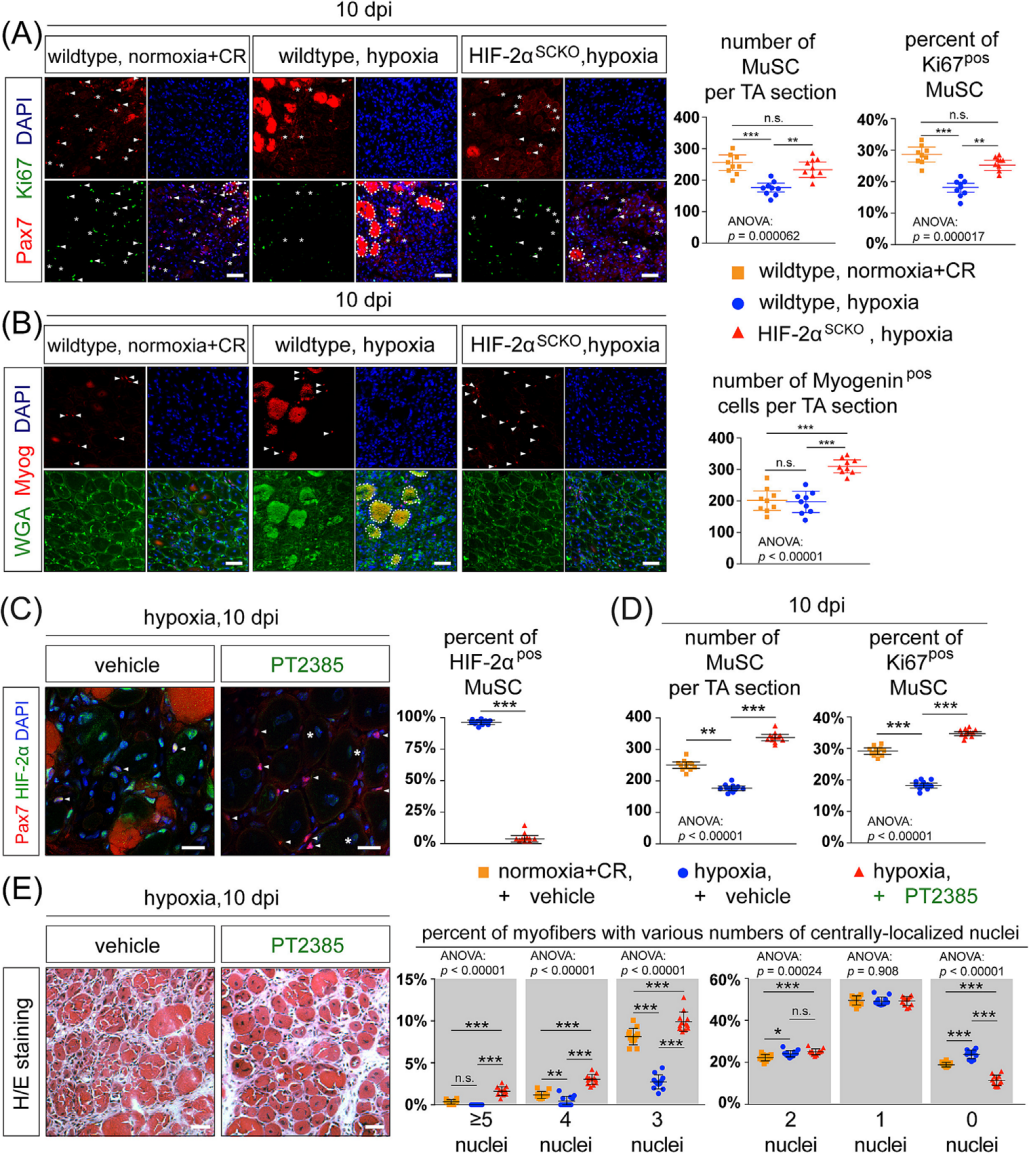
Rescue of impaired proliferation and differentiation of MuSCs and regeneration failure under chronic hypoxia by targeting HIF‐2A through MuSCs‐specific HIF‐2A ablation or the administration of HIF‐2A inhibitor PT2385. (A) Left: Representative IM images of Pax7 and Ki67 (a proliferation marker) on TA muscle cross‐sections at 10 dpi from wildtype and HIF‐2α^SCKO^ mice under normoxia+CR and chronic hypoxia conditions, as described in Figure 1. Arrowheads: Pax7^pos^/Ki67^pos^ MuSCs. Asterisks: Pax7^pos^/Ki67^neg^ MuSCs. Circled areas: Staining artefacts with a high affinity to mouse antibodies, which resemble degenerative myofibres. Scale bar: 50 μm. Right: Quantifications of MuSCs number per TA muscle cross‐section and percentages of Ki67^pos^ MuSCs in the total MuSCs pool (*n* = 9 per group, 5 males and 4 females). Conditions are the same as in the left panel. (B) Left: Representative IM images of Myogenin and WGA staining on TA muscle cross‐sections at 10 dpi from wildtype and HIF‐2α^SCKO^ mice under the normoxia+CR and chronic hypoxia conditions as described in Figure 1. Arrowheads: Myogenin^pos^ MuSCs. Circled areas: Suspected degenerative myofibres. Scale bar: 50 μm. Right: Quantifications of Myogenin^pos^ cell number per TA muscle cross‐section (*n* = 9 per group, 5 males and 4 females). Conditions are the same as in the left panel. (C) Left: Representative IM images of Pax7 and HIF‐2α on TA muscle cross‐sections at 10 dpi from *C57BL6/J* mice under chronic hypoxia. The TA muscles were treated with either vehicle or PT2385 (1.5 μg per TA muscle per injection) at 3–5 dpi. Notably, HIF‐2α is expressed in both Pax7^pos^ and Pax7^neg^ cells under hypoxia. PT2385 repressed HIF‐2α expression in Pax7^pos^ MuSCs (arrowheads) but low levels of HIF‐2α still existed around centralized myonuclei (asterisks) within newly formed myofibres. Scale bar: 50 μm. Right: Quantifications of percentages of HIF‐2α^pos^ MuSCs in the total MuSCs pool (*n* = 12 per group, 6 males and 6 females). Conditions are the same as in the left panel. (D) Quantifications of MuSCs number per TA muscle cross‐section (left) and percentages of Ki67^pos^ MuSCs in the total MuSCs pool (right) (*n* = 12 per group, 6 males and 6 females) at 10 dpi from wildtype mice under normoxia+CR or chronic hypoxia conditions and treated with either vehicle or PT2385. (E) Left: H&E staining of TA muscle cross‐section at 10 dpi from the chronic hypoxia models treated with either vehicle or PT2385. Scale bar: 50 μm. Right: Quantifications of myofibres with different numbers of centrally‐localized myonuclei per TA muscle cross‐section at 10 dpi (*n* = 12 per group, 6 males and 6 females). Statistical analysis legend: In panels (A), (B), (D), and (E), one‐way ANOVA was conducted to assess the equality of means among three groups. When ANOVA *P*‐value is less than 0.05, post hoc Tukey HSD tests were performed to determine significant differences between pairs of groups. The results of these post hoc tests have been denoted between the respective pairs of groups. In the panel (C) right, Student's *t*‐test was conducted to assess the equality of means between vehicle and PT2385 groups. The results of statistical tests: ***P‐value < 0.001, **P‐value < 0.01, *P‐value < 0.05, n.s., not significant. Error bars represent standard deviations (SD).

At 10 dpi, injured TA muscles of *C57BL/6J* (wildtype) mice exposed to chronic hypoxia contained decreased number of total and proliferative (Pax7^pos^/Ki67^pos^) MuSCs compared with the normoxia group (Figure 3A). In contrast, HIF‐2α^SCKO^ mice exposed to identical chronic hypoxia had significantly more total and proliferative MuSCs (Figure 3A). Thus, MuSCs‐specific HIF‐2α ablation is beneficial for MuSCs proliferation under chronic hypoxia. For hypoxia‐exposed wildtype muscles, we noticed frequent incidences of immunostaining artefacts that are commonly associated with degenerative myofibres, characteristic of an earlier regeneration stage under normoxia (Figure 3A, circulated areas in the ‘wildtype, hypoxia’ group) (S20).

We also examined the expression of Myogenin in MuSCs as an indicator of their myogenic potential. At 10 dpi, chronic hypoxia did not affect the number of Myogenin^Pos^ MuSCs (Figure 3B). Interestingly, damaged TA muscles in HIF‐2α^SCKO^ mice showed an increase in Myogenin^Pos^ MuSCs (Figure 3B) as well as noticeable enhancement of myofibre structures (Figure 3B: left, WGA staining), compared with the wildtype mice exposed to identical chronic hypoxia.

The above findings from HIF‐2α^SCKO^ mice prompted us to investigate the potential therapeutic effects of the HIF‐2α inhibitor PT2385 in the context of muscle regeneration under chronic hypoxia. As proof of principle, we administrated PT2385 through intramuscular injections at 3–5 dpi in mice exposed to chronic hypoxia (Figure S1). At 10 dpi, HIF‐2α expression was detected in both Pax7^pos^ MuSCs and non‐myogenic cells, but PT2385 treatments noticeably reduced HIF‐2α levels (Figure 3C: left), significantly lowering the percentages of HIF‐2α^pos^ MuSCs (Figure 3C: right). Interestingly, low levels of HIF‐2α expression were still evident around the centralized myonuclei within regenerating myofibres (Figure 3C: left).

Notably, HIF‐2α antagonism significantly improved MuSCs proliferation under chronic hypoxia. At 10 dpi, the numbers of total and proliferative MuSCs both increased with PT2385 treatment under hypoxia, even surpassing the corresponding levels in control mice under normoxia conditions (Figure 3D).

The prominent effect of PT2385 on MuSCs proliferation also led to significant improvements in myogenic differentiation under chronic hypoxia, as evidenced by the increases in the numbers of centrally‐localized myonuclei at 10 dpi (Figure 3E).

### PT2385 improves muscle mass and function recovery while preventing injury‐associated fibrosis and fat infiltration under chronic hypoxia

**Figure 4:**
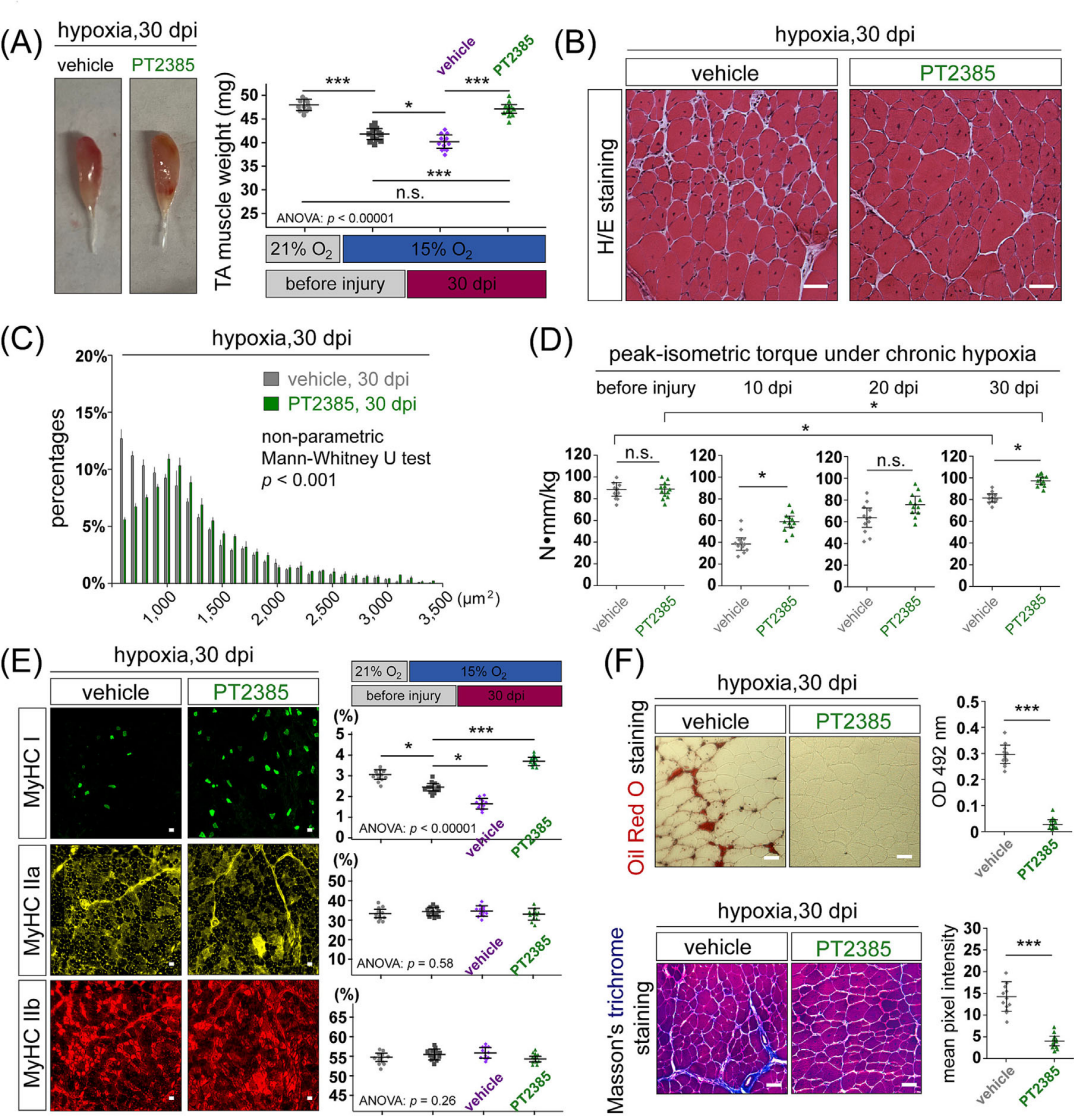
Effects of PT2385 on the recovery of muscle mass, morphology, and strength after muscle regeneration under chronic hypoxia. (A) Left: Images of dissected TA muscles from mice in the vehicle or PT2385 treatment groups at 30 dpi. Right: TA muscle weight comparison (*n* = 12, 6 males and 6 females) between mice under nomoxia+CR (light grey) and hypoxia conditions (dark grey) at the end of the 2‐week hypoxia adaptation stage (before injury) and from mice at 30 dpi after muscle regeneration under hypoxia but treated with either vehicle (purple) or PT2385 (green). (B) H&E staining of TA muscle cross‐section at 30 dpi from the chronic hypoxia models treated with either vehicle or PT2385. Scale bar: 50 μm. (C) Distribution of myofibre cross‐sectional areas in the conditions as the panel (B). (D) Peak‐isometric torques of the TA/EDL muscle group from the mice under the above conditions (*n* = 12 per group, 6 males and 6 females) were measured before CTX injury and at 10‐, 20‐, and 30‐dpi. (E) Left: Representative IM images of myosin heavy chain (MyHC) I, IIa, and IIb in TA muscles from the vehicle or PT2385 treatment groups at 30 dpi. Scale bar: 50 μm. Right: Quantification of the percentages of myofibres positively stained for MyHC I, IIa, and IIb in conditions described in the panel (A) (*n* = 12 per group, 6 males and 6 females). (F) Representative staining images of TA muscle cross‐sections from the vehicle or PT2385 treatment groups at 30 dpi. Upper panels: Oil Red O staining. Lower panels: Masson's trichrome staining. Scale bar: 50 μm. Right panels: Quantification of Oil Red O staining and Masson's trichrome staining in the left panels (*n* = 12 per group, 6 males and 6 females). Statistical analysis legend: In panels (A) and (E), one‐way ANOVA was conducted to assess the equality of means among four groups. When ANOVA *P*‐value is less than 0.05, post hoc Tukey HSD tests were performed to determine significant differences between pairs of groups. The results of these post hoc tests have been denoted between the respective pairs of groups. In panels (D) and (F), Student's *t*‐tests were conducted to assess the equality of means between specific two groups denoted by the crossbars. The results of t‐tests: ***P‐value < 0.001, **P‐value < 0.01, *P‐value < 0.05, n.s., not significant. Error bars represent standard deviations (SD). In the panel (C), the means of vehicle and PT2385 treatment groups were compared by Mann–Whitney *U* test.

Next, we assessed the impacts of PT2385 treatment on the outcome of muscle regeneration under chronic hypoxia. At 30 dpi, TA muscles treated with PT2385 were noticeably bigger than the control (Figure 4A: left). The TA muscle mass was significantly reduced after chronic hypoxia adaptation, which worsened after regeneration (Figure 4A: right). In contrast, PT2385 treatment increased TA muscle mass after regeneration, reaching a level comparable to that of normal regenerated muscle under normoxia (Figure 4A: right). H&E staining and myofibre CSA confirmed the enlargement of regenerated myofibre calibres in PT2385‐treated muscle compared with the control (Figures 4B,C).

We measured the peak‐isometric torques of the TA/EDL muscle group at four time points during muscle regeneration under hypoxia. Before CTX injury, the vehicle and PT2385 groups exhibited similar levels of muscle strength (Figure 4D). However, during the recovery of muscle strength (10, 20, and 30 dpi), PT2385‐treated muscles demonstrated noticeably accelerated strength recovery compared with the control (significant at 10 and 30 dpi; Figure 4D). Notably, after 30 days of regeneration under hypoxia, the control muscles still did not fully recover their strength; in contrast, PT2385‐treated muscles not only fully recovered but also displayed increased torque production compared with the state before muscle damage (Figure 4D).

Furthermore, we assessed the impacts of chronic hypoxia and PT2385 treatments on myofibre‐type composition. Hypoxia adaptation reduced the percentage of type I myofibres in TA muscles, and this reduction was further exacerbated after muscle regeneration under hypoxia (Figure 4E). Interestingly, PT2385 treatments led to an increase in type I myofibres (Figure 4E). There were no significant alterations in the percentages of type IIb or type IIa myofibres (Figure 4E).

COPD Patients have increased muscle fibrosis and ectopic fat accumulation in skeletal muscles.^9^, ^22^ At 30 dpi, Oil Red O staining revealed the widespread presence of triglyceride deposits between myofibres in vehicle‐treated muscles, which were absent in PT2385‐treated muscles (Figure 4F: upper). Masson's trichrome staining showed higher levels of fibrotic collagen deposition within inter‐myofibril spaces in vehicle‐treated muscles compared with PT2385‐treated muscles (Figure 4F: lower), indicating reduced muscle fibrosis upon PT2385 treatment.

Our previous study demonstrated that long‐term deficiency of HIF‐2α in MuSCs leads to muscle hypertrophy but MuSCs depletion due to continuous activation and myogenic differentiation.^15^ This can be prevented by a transient HIF‐2α antagonism regime in the early regeneration stages, as employed in this study. Regeneration under chronic hypoxia resulted in a reduction of MuSCs pool at 30 dpi, which was rescued by PT2385 (Figure S2). This suggests that muscle damage under chronic hypoxia may impair the long‐term repairing potential due to diminished MuSCs maintenance. Importantly, HIF‐2α inhibitor PT2385 may help to restore a functional MuSCs pool through its beneficial effects during muscle regeneration.

### A HIF‐2A‐dependent chronic hypoxia signature in myoblasts includes ACE, a direct target of HIF‐2A

**Figure 5:**
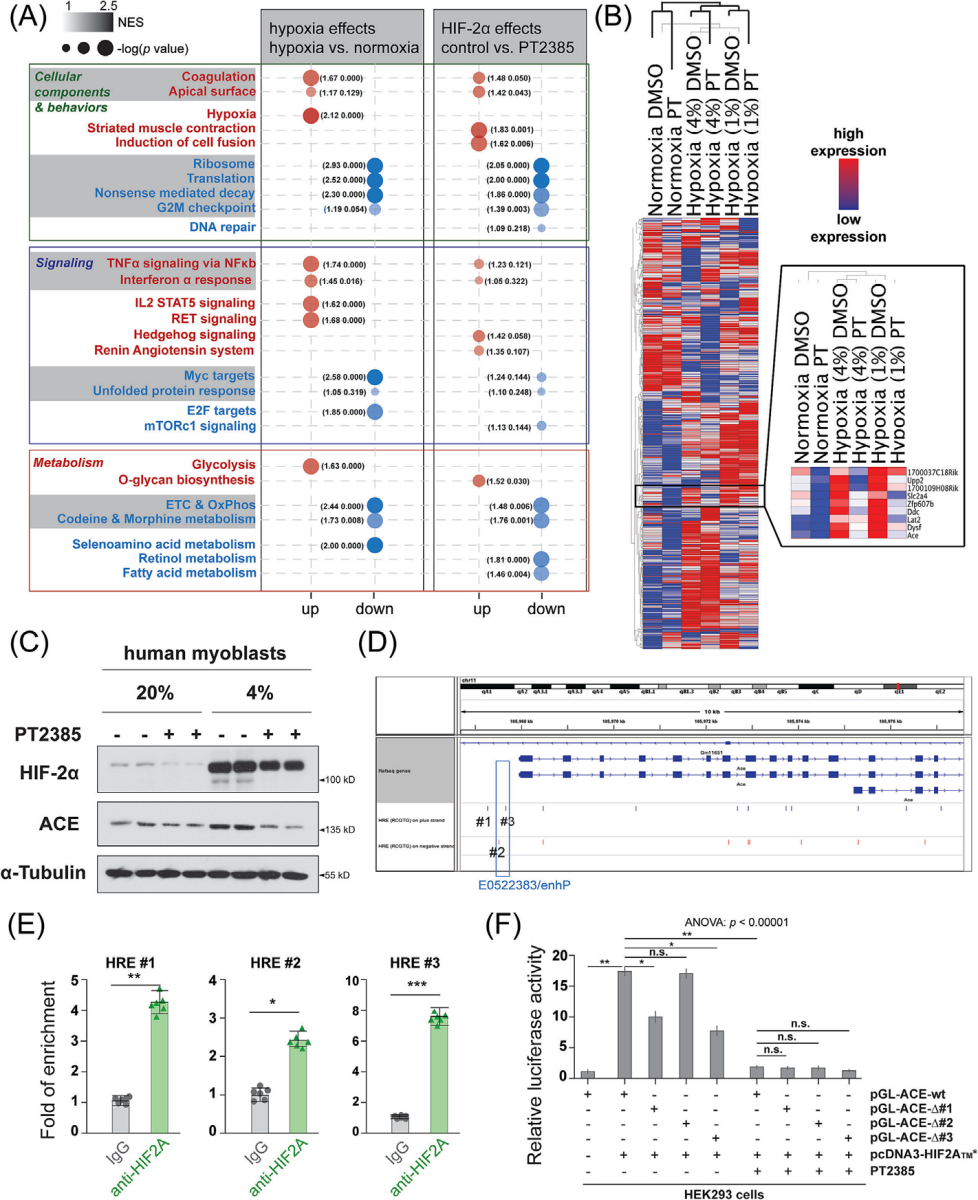
A HIF‐2A‐dependent chronic hypoxia signature in myoblasts includes ACE as a direct target of HIF‐2A. (A) Summary of results from gene set enrichment analysis (GSEA). Gene expression values from six RNA‐seq conditions were analysed: Three cell conditions (20% *p*O_2_ normoxia, 4% *p*O_2_ mild hypoxia, and 1% *p*O_2_ severe hypoxia) intersecting with two drug treatment conditions (0.1% DMSO vehicle and 1 μM PT2385). GSEA was conducted to compare hypoxia effects and HIF‐2α effects. The top three gene sets shared between the two comparisons (shaded) or specific to one comparison (non‐shaded) were listed on the left if the normalized *P*‐values are less than a threshold of 0.25 (from leading edge analysis) and grouped into categories of ‘Cellular Components & Behaviors’, ‘Signalling’, and ‘metabolism’. Gene sets enriched in upregulated (red) or downregulated (blue) genes in the comparisons (refer to the column titles) were separated and represented by circles. The shades of the circles indicate the normalized enrichment index (NES) in GSEA, while the sizes of the circles indicate the negative log_10_ of *P*‐values. (B) Unsupervised hierarchical clustering and heat maps of gene expression in six RNA‐seq conditions. The black box outlines a gene signature induced by HIF‐2α. The extended box is the zoom‐in of the gene signature with detailed information. (C) Western blotting of HIF‐2α, ACE, and α‐tubulin (a loading control) in human myoblasts cultured under 20% *p*O_2_ ‘normoxia’ or adapted to 4% *p*O_2_ hypoxia conditions for 2 weeks. Under both conditions, human myoblasts were treated with vehicle (‘‐’ sign) or PT2385(‘+’ sign; 200 nM) for 48 h. (D) A screenshot of the integrated genome viewer displaying the *ACE* spanned genomic region on chromosome 11 in the mouse genome. Hypoxia response elements (HREs) on the plus (blue) and minus (red) strands were depicted by bars. Three HREs close to the *ACE* promoter were labelled and used in further experiments. The region annotated as ‘E0522383/enhP’ was depicted by a box. (E) ChIP‐qPCR results (*n* = 6) show the relative enrichments of HIF‐2α at the above three HRE regions. (F) Luciferase assay results (*n* = 20) show the activities of the wildtype (wt) *ACE* promoter and mutated versions (with targeted mutations in HREs #1, #2, or #3) under the conditions of HIF‐2α overexpression and/or PT2385 treatment. Statistical analysis legend: In the panel (E), Student's *t*‐tests were conducted to assess the equality of means between IgG control group and the anti‐HIF2A ChIP group. The results of the *t*‐tests are denoted by the crossbars: ***P‐value < 0.001, **P‐value < 0.01, *P‐value < 0.05, n.s., not significant. In the panel (F), one‐way ANOVA was conducted to assess the equality of means among nine groups. As ANOVA *P*‐value is less than 0.05, post hoc Tukey HSD tests were performed to determine significant differences between pairs of groups. The results of these post hoc tests have been denoted between the respective pairs of groups: ***P‐value < 0.001, **P‐value < 0.01, *P‐value < 0.05, n.s., not significant. Error bars represent standard deviations (SD).

Next, we investigated the impacts of chronic hypoxia‐stabilized HIF‐2α on the transcriptome using a murine myoblast model. MuSCs‐derived myoblasts were initially cultured under normal 20% *p*O_2_ conditions and then acclimatized to three separate oxygen tensions (20% *p*O_2_ normoxia, 4% *p*O_2_ hypoxia, and 1% *p*O_2_ hypoxia). For each condition, myoblasts were treated with either PT2385 or a vehicle solution (0.1% DMSO). The 4% *p*O_2_ hypoxia and 1% *p*O_2_ hypoxia conditions were chosen to mimic moderate and severe hypoxic conditions in limb muscles, respectively.^21^ We conducted RNA‐seq and GSEA to examine the effects of hypoxia (by comparing 4% and 1% hypoxia conditions with the normoxia condition in the vehicle groups) and HIF‐2α‐specific effects (by comparing PT2385 conditions with vehicle conditions under 4% and 1% hypoxia; Figure 5A). Interestingly, there were both overlapping and distinct effects of hypoxia and HIF‐2α. For instance, both hypoxia and HIF‐2α promoted the upregulation of genes associated with categories such as ‘coagulation’, ‘apical surface structures’, ‘TNF signalling via NFκB’, and ‘Interferon α response’. However, hypoxia‐induced effects also included ‘IL‐2 STAT5 signalling’, ‘RET signalling’, and ‘Glycolysis’, which were not observed in HIF‐2α‐dependent effects. Conversely, HIF‐2α affected ‘Striated muscle contract’, ‘Induction of cell fusion’, ‘Hedgehog signalling’, ‘Renin‐Angiotensin system’, and ‘O‐glycan biosynthesis’, which were absent in hypoxia‐dependent effects. In a holistic perspective that focuses on the shared gene categories between hypoxia and HIF‐2α, a predominance of downregulated expression levels (shaded blue) was noted as opposed to upregulated levels (shaded red; Figure 5A). This implies that HIF‐2α‐mediated transcriptional regulation contributes mainly to gene expression repression under chronic hypoxia.

The unsupervised hierarchical clustering grouped conditions under the same *p*O_2_ levels together, suggesting that the overall impacts of hypoxia surpass the effects attributed to HIF‐2α (Figure 5B). We identified a gene signature that depends on the transcriptional activity of HIF‐2α (Figure 5B: extended box). This signature includes the *ACE* gene, which encodes a key enzyme in the ‘Renin‐Angiotensin system’ (RAS). We focused on ACE in subsequent investigations. Stabilization of HIF‐2α was evident in human myoblasts cultured under 4% *p*O_2_ for 2 weeks (Figure 5C). For both normoxic and hypoxic conditions, PT2385 treatment led to noticeable reductions in the expression levels of HIF‐2α and ACE, confirming ACE is a bona fide target of HIF‐2α in both humans and mice.

Three HIF‐2α binding motifs (HRE:5′‐RCGTG‐3′) are present within the *ACE* promoter (Figure 5D). Two of these motifs (HRE #2 and HRE #3) are localized within a known enhancer/promoter region (E0522383) in the mouse genome. To probe the binding of HIF‐2α to these regions containing HRE motifs, we performed ChIP‐qPCR using mouse myoblasts exposed to chronic hypoxia (2% *p*O_2_ for 2 weeks; Figure 5E). The results revealed that HIF‐2α bound to all three genomic regions under chronic hypoxia, with the region surrounding HRE#3 displaying the most pronounced enrichment.

We performed luciferase assays to further confirm the transactivation activity of HIF‐2α on the *ACE* promoter. Co‐transfection of HIF‐2α‐overexpressing and *ACE* promoter‐containing plasmids resulted in a significant increase in luciferase activity, which was inhibited by PT2385 and diminished by mutations in HRE#1 and HRE#3 sequences (Figure 5F). Thus, these two HREs may mediate the transactivation of mouse *ACE* promoter by HIF‐2α.

### HIF‐2A augmented ACE expression in muscle stem cells under chronic hypoxia and lisinopril enhanced muscle stem cells proliferation during muscle regeneration under chronic hypoxia

**Figure 6:**
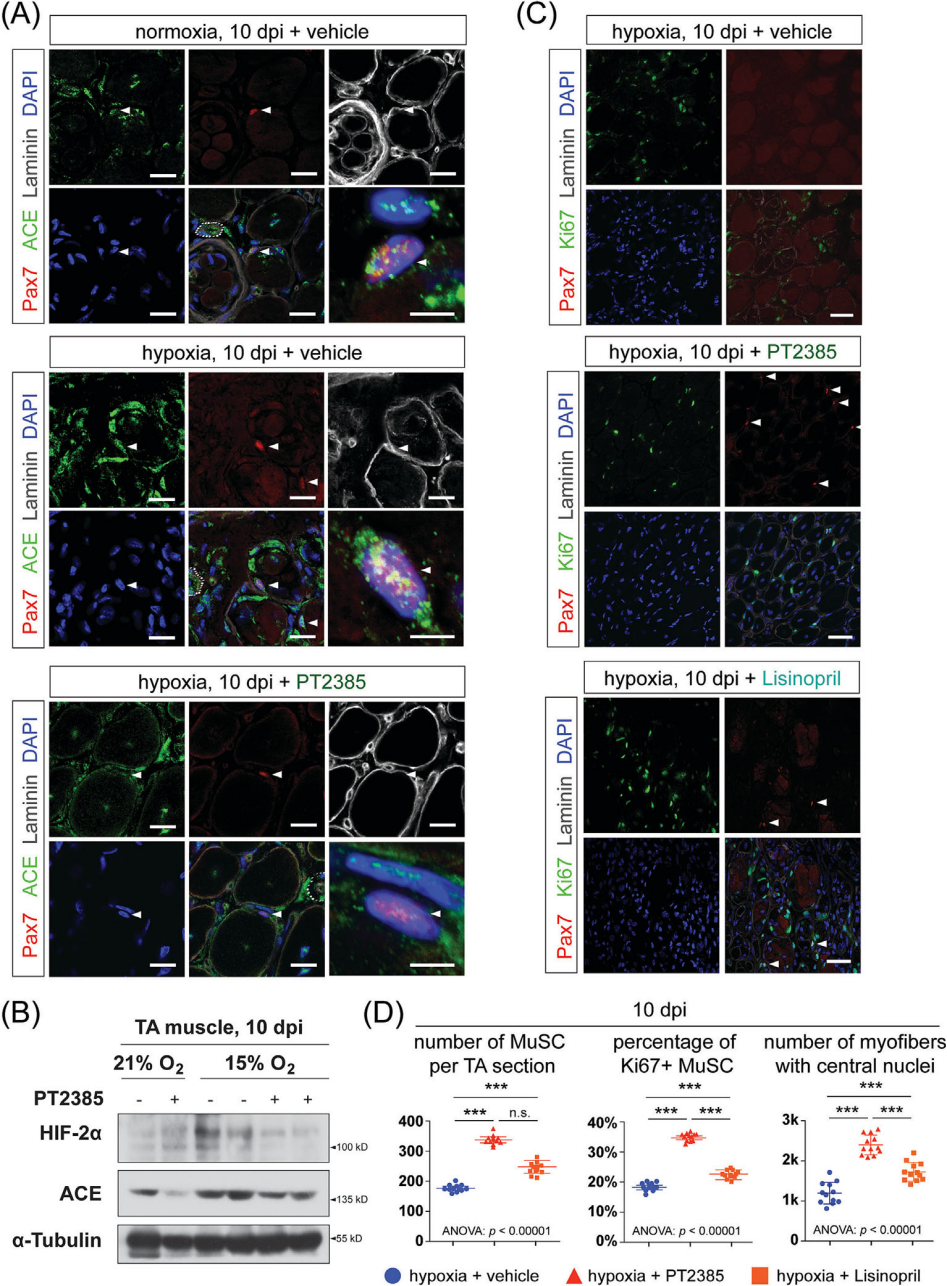
HIF‐2A enhances ACE expression in MuSCs and the ACE inhibitor lisinopril increased MuSCs proliferation during muscle regeneration under chronic hypoxia. (A) Representative IM images of Pax7, ACE, and laminin B2 on TA muscle cross‐sections at 10 dpi from mice in the normoxia+CR group and the chronic hypoxia models treated with either vehicle or PT2385, as described in panels (D) and (E) of Figure 3 (*n* = 12 per group, 6 males and 6 females). The lower right panel in each image group shows a zoom‐in image of the indicated Pax7^pos^ MuSCs (arrowhead) with higher magnification. Circled areas: Blood vessels. Scale bar: 10 μm. *(B)* Western blotting of HIF‐2α, ACE, and α‐tubulin in tissue lysates of CTX‐injured TA muscles at 10 dpi from mice under normoxia (21% *p*O_2_) or chronic hypoxia (15% *p*O_2_) treated with either vehicle (‘−’ sign) or PT2385 (‘+’ sign; 1.5 μg per TA muscle per injection) at 3–5 dpi. (C) Representative IM images of Pax7, Ki67, and laminin B2 on TA muscle cross‐sections at 10 dpi from the chronic hypoxia models treated with vehicle, PT2385 or lisinopril (1 μg; IC_50_ = 4.7 nM) at 3–5 dpi (*n* = 12 per group, 6 males and 6 females). Arrowheads: Pax7^pos^/Ki67^pos^ proliferative MuSCs. Scale bar: 50 μm. (D) Quantification of MuSCs number per TA muscle cross‐section (left), percentages of Ki67^pos^ MuSCs in the total MuSCs pool (middle), and numbers of myofibres with centrally‐localized myonuclei (right) at 10 dpi (*n* = 12 per group, 6 males and 6 females). Conditions are the same as in the panel (C). Statistical analysis legend: In the panel (D), one‐way ANOVA was conducted to assess the equality of means among ‘hypoxia+vehicle’ (0.1% DMSO), ‘hypoxia+PT2385’, and ‘hypoxia+lisinopril’ groups. As ANOVA *P*‐values are less than 0.05, post hoc Tukey HSD tests were performed to determine significant differences between pairs of groups. The results of these post hoc tests have been denoted between the respective pairs of groups: ***P‐value < 0.001, **P‐value < 0.01, *P‐value < 0.05, n.s., not significant. Error bars represent standard deviations (SD).

We conducted additional investigations to understand the biological significance of the HIF‐2α/ACE axis in the context of hypoxia‐induced regeneration defects. To confirm the presence of the HIF‐2α/ACE axis *in vivo*, we evaluated ACE expression levels in MuSCs (10 dpi) across different conditions: normoxia, hypoxia, and hypoxia with PT2385 treatment (Figure 6A). At 10 dpi, ACE signals can be detected encompassing Pax7^pos^ MuSCs and other cell types. Chronic hypoxia induced a global increase in ACE expression, including its expression in MuSCs (Figure 6A: middle). Remarkably, under the same hypoxic condition, PT2385 treatment attenuated ACE expression in MuSCs (Figure 6A: lower), confirming the HIF‐2α/ACE axis in MuSCs under hypoxia. The ACE protein levels in whole‐muscle lysates essentially corroborated the ACE immunostaining signals (Figure 6B). Notably, ACE exhibited discernible expression in centrally‐localized myonuclei, which emerged in PT2385‐treated hypoxic muscle at 10 dpi (Figure 6A: lower).

We then explored the implications of locally acting ACE on MuSCs proliferation—a key factor contributing to regeneration failure under chronic hypoxia. We administered ACE inhibitor lisinopril (IC_50_ = 5.6 nM) using the same drug administration regimen as for PT2385, to the CTX‐injured TA muscle in the chronic hypoxia models. Compared with the vehicle‐treated group, lisinopril elevated the number of total and Ki67^pos^ proliferative MuSCs at 10 dpi (Figure 6C,D: left and middle), albeit to a lesser extent than observed with PT2385. Notably, the increase in MuSCs proliferation in the PT2385 and lisinopril treatment groups correlated with an improvement in muscle regeneration, as evidenced by the significant increases in myofibres with centrally‐localized myonuclei, compared with the vehicle group (Figure 6C,D: right).

## Discussion

In this study, we employed a 6‐week normobaric hypoxic air exposure as a surrogate model of chronic hypoxia conditions in COPD patients. Previous studies have mainly focused on short‐term hypoxia exposure or long‐term adaptation to high altitudes,^14^ which may not fully replicate COPD‐associated pathology. COPD patients experience prolonged mild hypoxemia (93–95% SpO_2_) without a compelling need for oxygen supplementation.^18^ Investigating the health impacts of such conditions requires experimental settings that can simulate chronic moderate hypoxia. Prolonged hypobaric hypoxia at high altitudes does involve adaptative responses to chronic hypoxia, but it fails to mimic the gas exchange failure associated with COPD. The reduced air pressure at high altitudes facilitates CO_2_ diffusion from the pulmonary arteries to the alveoli, which contrasts with the hypercapnic condition in COPD patients. The experimental conditions in the present study also have limitations in this regard as the mixed hypoxic air was supplemented with nitrogen gas instead of CO_2_, which cannot fully replicate COPD hypercapnia.

The normobaric hypoxia models in this study showed a reduction of myoglobin levels in limb muscles, recapitulating the condition in COPD patients.^23^ Myoglobin plays a crucial role in oxygen storage, buffering, and diffusion within myofibres (S21). Its reduction implies that chronic hypoxia not only affects oxygen supply but also diminishes oxygen transport and storage capacities in skeletal muscle.

Despite different nature, both normobaric hypoxia investigated in this study and the hypobaric hypoxia^14^ impaired muscle regeneration, stressing the detrimental effect of chronic hypoxia on muscle repair. The hypoxic effect on MuSCs cannot be solely attributed to energy deficits. Numerous studies have shown that adaptative responses triggered by HIF‐1α stabilization or other hypoxia‐related mechanisms in the early regenerative stage promote a bioenergetic switch, enhancing MuSCs proliferation through increased anaerobic glycolysis (,^24^, ^25^, ^26^S22). Indeed, the exacerbated outcomes observed after HIF‐1α ablation or its inhibition underscore the essential role of HIF‐1α in muscle regeneration under hypoxia.

This study provides strong evidence to support HIF‐2α as a key contributor to the detrimental effects of chronic hypoxia on muscle regeneration. Chronic hypoxia led to an increase in HIF‐2α expression in proliferating MuSCs and a concomitant decrease in their proliferation rate. This aligns well with our previous finding that HIF‐2α overexpression impedes MuSCs proliferation.^15^ Here, we provided the first view of transcriptomic alterations in myogenic cells associated with HIF‐2α activity and chronic hypoxia. Collectively, HIF‐2α appears to mediate the inhibitory effects of chronic hypoxia on gene expression, particularly through the repression of genes related to ribosome function, protein translation, unfolded protein response, and ETC/OxPhos. Notably, these changes are closely linked to myofibre atrophy,^27^, ^28^, ^29^ suggesting a potential contribution of HIF‐2α to cachexia beyond the stem cell compartment. Additionally, HIF‐2α represses the expression of genes associated with DNA repair, E2F transcription factors, mTORc1 signalling, and retinol/fatty acid metabolism under chronic hypoxia, a contrast not observed in the comparison between hypoxia versus normoxia. These unique effects highlight the intricacy of HIFα's role under hypoxia (S11). For instance, while HIF‐1α mediates the hypoxia‐induced shift towards anaerobic glycolysis, HIF‐2α notably assumes an opposite role, as evidenced by the substantial rise in glycolytic rates upon HIF‐2α inhibition.

HIF‐2α activates expression of genes associated with coagulation, apical surface structures, TNFα/NF‐κB signalling, and interferon α response, which have been implicated in impaired MuSCs proliferation.^30^, ^31^ Interestingly, HIF‐2α also induces genes related to muscle contraction and fusion under hypoxia, in contrast to the inhibitory effect of HIF‐2α overexpression on myogenic differentiation under normoxia.^15^ This might be attributed to the robust pro‐proliferative effect of PT2385 under hypoxia, potentially counteracting the spontaneous myogenic differentiation program that occurs during primary myoblast culture.

Intriguingly, HIF‐2α inhibitor PT2385 resulted in a significantly improved regeneration outcome compared with HIF‐2α genetic ablation in MuSCs, even surpassing the levels observed under normoxia. This suggests functions of HIF‐2α in non‐myogenic lineages. Notably, HIF‐2α ablation and its inhibition showed comparable effects on regeneration under normoxia in our previous study.^15^ The exceptional efficacy of PT2385 in this study further suggests that chronic hypoxia introduces additional impediments to muscle regeneration beyond the stem cell compartment delineated by *Pax7* ^*cre*^.

Endothelial cells (EC) and macrophages may contribute to these additional adverse effects. HIF‐2α is highly expressed in endothelium (S23). Within skeletal muscle, MuSCs and EC are in close juxtaposition and interact with each other through NOTCH receptors and DLL4 ligands, which promotes MuSCs quiescence and self‐renewal (S24–25). Secreted factors from both EC and MuSCs couple regenerative angiogenesis and myogenesis in a temporally and specially coordinated manner,^32^ which are compromised under hypoxic/ischaemic conditions.^33^ Therefore, the disengagement of endothelial proliferation and MuSCs activation due to HIF‐2α stabilization could diminish proliferation potential and/or induce premature myogenesis in hypoxic muscle, in alignment with the observations in this study. Macrophages also play a pivotal role in muscle regeneration (S26). HIF‐2α stabilization in macrophages favours polarization towards M2 lineages^34^ and promotes anti‐inflammatory cytokine release, culminating in immune‐suppressive and immune‐privilege effects.^35^ Thus, aberrant macrophage polarization under chronic hypoxia could attenuate MuSCs activation/proliferation. Further investigations should focus on deciphering the intricate crosstalks between MuSCs and other cell populations in regenerative muscle under chronic hypoxia, which would uncover new therapeutic candidates for COPD and CHF.

The identification of HIF‐2α transactivating ACE represents a novel yet unsurprising discovery. *Epas1* ^+/−^ heterozygous mice are protected against chronic hypoxia‐induced pulmonary hypertension, showing substantial reductions in ACE expression in their lungs and heart (S27). Intriguingly, pulmonary hypertension is associated with COPD (S28) and HIF‐2α plays an essential role in driving pulmonary hypertension.^36^ The newfound HIF‐2α/ACE axis provides a mechanistic framework elucidating the disease association.

ACE is a key enzyme in RAS, responsible for generating bioactive Angiotensin II. Well known as a vasoconstrictor, Angiotensin II also acts as a potent inducer of muscle atrophy^37^ (S29). Increased angiotensin II have been correlated with cachexia in patients with COPD, CHF, and chronic kidney disease (CKD) (S30–31). Emerging evidence highlights the importance of local hypoxia and tissue‐activated RAS in the progression of the above chronic disorders (S32). The present study reinforces this notion and offers mechanistic insights, connecting the local ACE increase with the transcription output of HIF‐2α.

While the exact mechanism of Angiotensin II in cachexia remains incompletely understood, its atrophic effect in non‐injured muscles appears to rely on oxidative stress (S29, S33) and cytokines IGF‐1 (S34) and IL‐6 (S35). ACE may exert both direct and indirect influences on regenerative muscle. Under chronic hypoxia, ACE overproduction expectedly sustain arterial constraints and exacerbate the local hypoxic milieu. Additionally, MuSCs‐derived ACE could directly impede MuSCs proliferation through its negative impact on MuSCs.^38^ The exact contributions necessitate further investigation using cell‐type specific genetic tools.

Lisinopril is a widely‐prescribed ACE inhibitor (ACEi) for hypertension and heart failure. Transient local administration of lisinopril led to favourable outcomes under hypoxia, supporting the involvement of HIF‐2α/ACE axis in hypoxia‐associated regeneration failure. This aligns with known beneficial benefits of ACEi or AT1R antagonists for COPD^39^ and CHF patients.^40^ Compared with lisinopril, PT2385 yielded notably more pronounced beneficial effects in the same hypoxia model, suggesting the involvement of other HIF‐2α targets in hypoxia‐associated regeneration failure. These results also emphasize the potential of HIF‐2α as a promising therapeutic target for COPD, addressing both COPD‐associated cachexia and pulmonary hypertension.

## Conflict of interest

The authors declare that there is potential for a conflict of interest associated with this work. The authors A.Y. and H.Y. are employed by HAWA Therapeutics, LLC, a small business that operates in a related field. While utmost care has been taken to ensure the objectivity, transparency, and integrity of the research findings, the authors acknowledge that their affiliation with HAWA Therapeutics, LLC might influence the interpretation of the results or discussions presented here. However, the authors affirm that the study was conducted with strict scientific standards and that the influence of their affiliation on the research process and outcomes was kept to a minimum. This conflict‐of‐interest statement is provided in the interest of transparency and to maintain the credibility and impartiality of the research conducted and reported herein.

## Supporting information


**Fig. S1.** (associated with Figure 1) Experimental design and timeline in this study.
**Figure S2.** (associated with Figure 4) Left: Representative IM images of Pax7 and Laminin B2 on TA muscle cross‐sections from the vehicle or PT2385 treatment groups at 30 dpi. Arrowheads: Pax7^pos^ MuSC. Scale bar: 50 μm. Right: Quantification of Pax7^pos^ MuSC on TA muscle cross‐sections in mice under nomoxia+CR (light grey) and hypoxia conditions (dark grey) at the end of the 2‐week hypoxia adaptation stage (before injury) and from mice at 30 dpi after muscle regeneration under hypoxia but treated with either vehicle (purple) or PT2385 (green). (*n* = 12 per group, 6 males and 6 females). Statistical analysis legend: in the right panel, one‐way ANOVA was conducted to assess the equality of means among normoxia, normoxia+CR, and hypoxia groups treated with vehicle or PT2385. When *p*‐value < 0.05 (rejection of the null hypothesis that all four groups have equal means), Post Hoc Tukey HSD tests were performed to determine significant differences between pairs of groups. The results of these Post Hoc tests have been denoted between the respective pairs of groups: ***: *p*‐value < 0.001, **: *p*‐value < 0.01, *: *p*‐value < 0.05, n.s.: not significant. Error bars represent standard deviations (SD).

## Data Availability

The raw RNA‐sequencing data generated in this study have been deposited in the Gene Expression Omnibus (GEO) repository under the accession number GSE243799.
