# Supplementary material for: Chronic hypoxia impairs skeletal muscle repair via HIF‐2α stabilization
Source: J Cachexia Sarcopenia Muscle. 2024 Feb 9;15(2):631–45. doi: 10.1002/jcsm.13436 (PMC10995261; doi:10.1002/jcsm.13436)
Supplement: Supplementary file 1 — Fig. S1. (associated with Figure 1) Experimental design and timeline in this study. Figure S2. (associated with Figure 4) Left: Representative IM images of Pax7 and Laminin B2 on TA muscle cross‐sections from the vehicle or PT2385 treatment groups at 30 dpi. Arrowheads: Pax7pos MuSC. Scale bar: 50 μm. Right: Quantification of Pax7pos MuSC on TA muscle cross‐sections in mice under nomoxia+CR (light grey) and hypoxia conditions (dark grey) at the end of the 2‐week hypoxia adaptation stage (before injury) and from mice at 30 dpi after muscle regeneration under hypoxia but treated with either vehicle (purple) or PT2385 (green). (n = 12 per group, 6 males and 6 females). Statistical analysis legend: in the right panel, one‐way ANOVA was conducted to assess the equality of means among normoxia, normoxia+CR, and hypoxia groups treated with vehicle or PT2385. When p‐value < 0.05 (rejection of the null hypothesis that all four groups have equal means), Post Hoc Tukey HSD tests were performed to determine significant differences between pairs of groups. The results of these Post Hoc tests have been denoted between the respective pairs of groups: ***: p‐value < 0.001, **: p‐value < 0.01, *: p‐value < 0.05, n.s.: not significant. Error bars represent standard deviations (SD). [file JCSM-15-631-s001.docx]

**SUPPLEMENTARY METHODS**

**Study Approval**

All animal studies were approved by the University of Georgia Institutional Animal Care and Use Committee (IACUC) and performed strictly following the guidelines.

**Animal Housing and Strains**

All animals were individually housed in a controlled environment at a room temperature of 23°C with 12:12-hour light-dark cycles. The following mouse strains were used in this study, all obtained from the Jackson Laboratory (Bar Harbor, ME): *C57BL6/J* (#000664), *Pax7^creERT2^* (#017763), *Hif2a^flox^* (#008407), and *Hif1a^flox^* (#007561). Equal numbers of males and females were used in all experiments.

To induce Cre activity in mice carrying *Pax7^creERT2^* allele, tamoxifen was administered intraperitoneally for three consecutive days (7 days before myofiber isolation) at a dosage of 100 mg/kg/day. Tamoxifen was prepared as a solution of 20 mg/mL in corn oil (Sigma #C8267).

**Additional Information about Chronic Hypoxia Treatment**

To ensure a stable environment and minimize disturbances, desiccant pouches were placed at the bottom of each chamber to absorb moisture, and ammonium filters and moisture filtration tanks were replaced every other day during the hypoxia treatment. During chamber cleaning/reconditioning (once a week), chambers were cleaned one at a time to avoid unnecessary disruptions to the ongoing experiments. To minimize stress during cleaning, the mouse inside was swiftly transferred to a spare metabolic chamber already filled with hypoxic air.

**Myofiber Culture & Hypoxia Chamber**

Individual myofibers were isolated from the extensor digitorum longus (EDL) muscle to facilitate the comparisons of results from this study and our previous study (Xie et al. *JCI* 2018). A modified protocol designed to minimize exposure to room air was employed in this study. Briefly, EDL muscle was carefully dissected and subsequently digested in a solution of 0.2% Type I Collagenase (Worthington) in DMEM at 37°C on a thermomixer for 1-1.5 hours. To maintain a hypoxic environment during the isolation process, a polished Pasteur pipette was connected to a regulated pressured tank containing hypoxia air (2% *p*O_2_). The Pasteur pipette was then inserted into the bottom of 15 mL tubes containing the EDL muscle in digestion. The controlled release of hypoxic air bubbles kept mildly disturbing the EDL muscle during digestion. Single myofibers were isolated from the digested EDL muscle by gently triturating the digested EDL muscle with polished Pasteur pipettes pre-coated with horse serum.

Myofibers were cultured in horse serum-coated 24-well plates in DMEM (4.5 g/L glucose) supplemented with 20% FBS, 1% sodium pyruvate, 1% chicken embryo-extract, and 1% penicillin-streptomycin. Two different oxygen conditions were used in myofiber culture: 20% *p*O_2_ ("normoxia", in regular cell culture incubators) or 4% *p*O_2_ (hypoxia, in a hypoxia chamber supplied with a mixed hypoxia air: 4% *p*O_2_ plus 5% CO_2_).

**Dual-Energy X-ray Absorptiometry (DEXA)**

DEXA was performed on a PIXImus Densitometer (GE Medical system) at UGA BioImaging Center. A default measurement protocol for bone density and soft tissue composition was used in this study.

**Bioimpedance Spectroscopy** (**BIA)**

BIA was performed using an ImpediVET single-channel tetrapolar device (Vet BIS1, ImpediMed) following a suggested protocol for rodents.

**Human Myoblast Culture**

The human myoblast cell line AB1079 was generously provided by Dr. Vincent Mouly from MyoBank (Thorley M. *Skeletal Muscle* 2016). AB1079 was isolated from the quadriceps muscle of a 38-year-old healthy human subject. The original biopsy was sourced anonymously from MyoBank, a biobank affiliated with EuroBioBank, which is authorized to distribute such human material by the French Ministry for Research (reference of the authorization AC-2019-3502).

Human myoblasts were cultured with Skeletal Muscle Cell growth medium (C-23160, PromoCell). When cells reached 70% confluence, cells were split and re-seeded at a density of 2x10^3^ cells per cm^2^.

**Intramuscular Injections and Drug Treatments**

To induce muscle injury, cardiotoxin (Sigma #C3987, 0.5 nmol, 100 µL) was injected into the TA muscle under anesthesia. For PT2385 or Lisinopril treatment, PT2385 (1.5 μg, 100 µL), Lisinopril (1.5 μg, 100 µL), or DMSO (0.1% solution, 100 µL) was injected into the TA muscle under anesthesia.

Notably, our observations indicate that injections (100 µL) into the TA muscle also elicit effects on the extensor digitorum longus (EDL) muscle, which is in close proximity to the TA muscle.

**Peak Isometric Torque Measurement**

Peak-isometric torque of the ankle dorsiflexors was assessed as previously described (Call JA, *J Appl Physiol*, 2011). Briefly, the left foot of anesthetized mice was placed on a foot plate attached to a servomotor (Model 300C-LR; Aurora Scientific). Two Pt-Ir electrode needles (Model E2-12; Grass Technologies) were inserted percutaneously on either side of the peroneal nerve. The ankle joint was secured at a 90° angle. Peak-isometric torque was achieved by varying the current delivered to the peroneal nerve at a frequency of 200 Hz and a 0.1-ms square wave pulse. Torques (N•mm) were normalized by the body mass (kg^-1^) to account for differences in body size.

**Estimation of Physical Activity by Beam-breaking Events**

Physical activities were measured by infrared beam manifolds (equipped with beams in x-, y-, and z-axes) set around the perimeter of the metabolic cages. A CLAMS system monitors 12 cages simultaneously. The bream-breaking counts per hour were averaged by calculating the geometric means of counts in x-, y-, and z-axes.

**Myoglobin Measurement**

Mouse myoglobin levels in TA muscle homogenates were measured using a Mouse Myoglobin ELISA Kit (Abcam, #ab210965) following the suggested protocol. Protein concentrations in muscle homogenates were measured by BSA assays (BioRad).

**Myofiber Cross-sectional Area (CSA) and Counts**

The automated measurements of myofiber cross-sectional areas on muscle IM images were performed as described in a previous publication (Mayeuf-Louchart A. et al. *Skeletal Muscle* 2018).

**Immunostaining**

For staining muscle sections, TA muscle sections were fixed with paraformaldehyde (PFA)/PBS (1%, 10 mins), quenched with glycine (50 mM, 10 mins), permeabilized with Triton X-100 (0.5%, 10 mins), blocked with Mouse on Mouse Blocking Reagent (Vector Lab) and 5% BSA/5% normal goat serum/PBS and incubated with primary antibodies: anti-Pax7 (1:5; DSHB #Pax7), anti-HIF2A (1:250; Novus Bio. #NB100-122), anti-HIF1A (1:250; Novus Bio. # NB100-105), anti-Ki67 (1:1,000; Abcam #ab15580), anti-Myogenin (1:50; DSHB #F5D), anti-ACE (1:1,000, Invitrogen #MA5-32741), anti-Laminin B2 (1:1,000; Millipore #05-206), anti-MyHC type I, IIA, or IIB (1:50, DSHB #BA-D5, #SC-71, #BF-F3) overnight at 4°C. Sections were washed in PBS/0.1% Tween-20, incubated with AlexaFluor-labeled secondary antibodies (1:200, 1 hr), washed, and mounted with DAPI-containing mounting medium (Life technologies). For WGA staining, sections were incubated with Wheat Germ Agglutinin conjugated with Alexa Fluor 647 (ThermoFisher #W32466) for 30 min before the final washing steps.

For staining myofibers, myofibers were fixed in PFA (4%, 10 mins), blocked in 5% BSA/5% normal goat serum/PBS, and incubated with primary antibodies: anti-Pax7 (1:50), anti-MyoD (1:250; Sigma #M6190), anti-HIF2A (1:250, Novus Bio. #NB100-122), and anti-HIF1A (1:250, Novus Bio. # NB100-105).

Mounted slides were imaged on a Zeiss LSM 710 confocal microscope.

**H/E Staining**

H/E staining was performed on muscle sections following an online protocol from the Treat-NMD consortium (SOP: MDC1A_M.1.2.004).

**Trichrome Staining**

Masson's Trichrome staining was performed on muscle sections following an online protocol from the Treat-NMD consortium (SOP: MDC1A_M.1.2.003).

The quantification of Masson's Trichrome staining was conducted following a modification of a previously published protocol (Xu L. et al. *Bio-Protocol* 2021). RGB color images of stained muscle sections were processed in Adobe Photoshop (version 24.7.0) using consistent settings: 1) in Image > Adjustments > Hue/Saturation, the Hue was adjusted to "+150"; and 2) in Image > Adjustments > Levels > Red channel, the median threshold was set to "0.1". Subsequently, in ImageJ2 (version 2.14.0/1.54f), the RGB color channels were separated, and measurements were taken from the "Red" channel.

**Oil Red O Staining**

Oil Red O staining was performed on frozen muscle sections following a previous publication (Bhullar A.S. et. al. *J. Cachexia Sarcopenia Muscle* 2020).

The quantification of Oil Red O (ORO) staining was performed following a modified protocol adapted from a previously published protocol (Du J. et al., *Adipocyte*, 2023). Briefly, five ORO-stained muscle sections, randomly selected from distinct levels within a muscle sample, were delineated using an ImmEdge® Hydrophobic PAP Pen. Subsequently, each section was immersed in 20 μL of 100% ethanol. The slides were then placed within a humidity box saturated with 100% ethanol and subjected to gentle shaking on an orbital shaker for 10 minutes. After the extraction of the ORO dye, 100% ethanol solutions from the five muscle sections were carefully aspirated, combined, and measured for absorbance at a wavelength of 492 nm.

**Mitochondrial Respiration Assay and Extracellular Acidification Assay**

Human myoblasts under hypoxia culture were plated on Seahorse XFe24 cell culture microplates (Agilent) and treated with drugs under the conditions described in the main text. Before the mitochondrial respiration assay, culture media were changed to Seahorse XF Base Medium supplemented with 2 mM glutamine, 1 mM sodium pyruvate, and 4.5 g/L glucose. Cells were placed in a non-CO_2_ incubator 1 hour before the Seahorse measurement. Oxygen consumption rates (OCR) were measured via the mitochondrial stress test assay kit (Agilent, 103015-100) with a Seahorse XFe24 analyzer following the manufacturer’s protocol. Before the extracellular acidification assay, culture media were changed to Seahorse XF Base Medium supplemented with 2 mM glutamine and 1 mM sodium pyruvate. Extracellular acidification rates (ECAR) were measured via the Glycolytic Rate Assay kit (Agilent, 103344-100) with a Seahorse XFe24 analyzer following the manufacturer’s protocol. Data was processed via Wave software (Agilent).

**Western Blotting**

Whole-cell and whole-muscle lysates were prepared by lysing cells/tissues in RIPA buffer supplemented with a proteinase inhibitor cocktail (1x). Protein concentration was quantified by BCA Protein Assays (Thermo Fisher Scientific). The membrane was blocked with 5% non-fat milk/TBST and probed with primary antibodies: anti-HIF2A (1:1,000, Novus #NB100-122), anti-ACE (1:1,000, Invitrogen #MA5-32741), and anti-alpha-Tubulin (1:5,000; Sigma #T6199), incubated with ECL reagents (Santa Cruz) and exposed to X-ray films.

**Luciferase Assay**

A mouse *ACE* promoter region (907 bp; -897 to +10 bp relative to TSS of ACE/NM_207624.6) was PCR amplified from genomic DNA (*C57BL/6* male) and cloned into pGL4.18 (Promega) to generate pGL-ACE wt. The three HRE sequences within the cloned region (HRE#1: "GCGTG", HRE#2: "GCGTG", and HRE#3: "ACGTG") were separately mutated into a "TGTCA" sequence by site-directed mutagenesis PCR to generate pGL-ACE-Δ#1, pGL-ACE-Δ#2, and pGL-ACE-Δ#3.

pGL-ACE wt, pGL-ACE-Δ#1, pGL-ACE-Δ#2, pGL-ACE-Δ#3 (100 ng/each) were separately co-transfected with pcDNA3-HIF2A^TM^ (Addgene) or a control empty pcDNA3 plasmid (100 ng) into HEK293 cells. pcDNA3.1-rLuc (50 ng) expressing Renilla luciferase was co-transfected as an internal transfection control. At 48 hours post-transfection, HEK293 cells were lysate with Luciferase Cell Lysis Buffer (NEB), followed by dual luciferase assay (ThermoFisher #16185).

**RNA-Sequencing and Gene Expression Calling**

Total RNAs were extracted from cultured mouse primary myoblasts exposed to either normoxic or hypoxic conditions by using RNA Extraction and Clean-up kits (Zymo Research). To reduce experimental variations and enhance the robustness of subsequent RNA-seq analysis, total RNAs were pooled from nine distinct myoblast cultures, which collectively represented three separate temporal replicates for each biological condition.

Total RNAs were sequenced on BGI DNBSEQ platforms. RNA reads in FastQ files were mapped to the mouse transcripts (mus_musculus.GRCm38.100.gtf) by TopHat2 installed in computer clusters at UGA GACRC.

**GSEA**

GSEA was performed using GSEA 4.0.3 (UC San Diego, Broad Institute) application with gene set databases: h.all.v2023.Hs.symbols.gmt [Hallmarks], c2.cp.kegg.v2023.1.Hs.symbols.gmt [curated], c2.cp.biocarta.v2023.1.Hs.symbols.gmt [curated], and c2.cp.wikipathway.v2023.1.Hs.symbols.gmt [curated]. Gene sets were permutated 2,000 times in each run of analysis. Gene sets with normalized enrichment score (NES) ≥ 1 and normalized *p* value ≥ 0.25 in hypoxia vs normoxia comparison or control vs. PT2385 comparison were collected. Normalized *p* value ≥ 0.25 was empirically determined by leading-Edge analysis. The top-ranked gene sets that are uniquely present in one type of comparison or shared by two types of comparisons were plotted with NESs and normalized *p* values in parentheses.

**Hierarchical Clustering**

Unsupervised hierarchical clustering was performed using the online GenePattern platform (genepattern.org).

**Chromatin Immunoprecipitation (ChIP) and qPCR**

Primary myoblasts under chronic hypoxia culture (4% *p*O_2_, 2 weeks) were resuspended in 200 μL lysis buffer (1% SDS, 10 mM EDTA, 50 mM Tris·HCl pH 8.1, 1x protease inhibitor cocktail) and sonicated at 4°C in a Bioruptor® Pico sonicator (Diagenode) using 15 on/off cycles of 30:30 seconds. The sheared chromatin (~300 bp) was 1:10 diluted in IP dilution buffer (50 mM HEPES-KOH pH7.5, 140 mM NaCl, 1 mM EDTA, 1% Triton X-100, 0.1% sodium deoxycholate, 0.1% SDS) and centrifuged at 20,000 xg, 4°C for 10 min. The supernatant was transferred to siliconized tubes and incubated with 2 μg HIF2A antibody (Novus #NB100-122) or rabbit IgG (Santa Cruz) on a rotating platform at 4°C overnight. PBS-washed Protein A Dynabeads^®^ (30 μL 50% slurry) were added to the chromatin/antibody mix and incubated on a rotating platform at 4°C for 4 hrs. All ChIP beads were sequentially washed with low salt, high salt, lithium, and TE buffer for 2 times/each and reverse-crosslinked with 1M NaCl for 6 hrs at 65°C. Samples were digested with 10 μg RNase A (37°C, 0.5 hr) and 20 μg Proteinase K (55°C, overnight). Immunoprecipitated genomic DNA was purified by phenol/chloroform/isoamyl alcohol (25:24:1) extraction followed by precipitation with 100% isopropanol, 0.3 M sodium acetate, and GlycolBlue at -20°C overnight. The following primers were used to quantify relative enrichment levels at HRE-flanking regions in the promoter of *ACE* in the mouse genome:

ACE_HRE#1_S: 5’-GGGGTTGTTGTAATCTCTAAGGC-3’

ACE_HRE#1_AS: 5’-ACACAGCTGTTCAACTCACA-3’

ACE_HRE#2_S: 5’-CAGGACAGCTAAACTTCCCG-3’

ACE_HRE#2_AS: 5’-TGGACTTAAGGTTGCCAGGT-3’

ACE_HRE#3_S: 5’-GTTATGCCAAGCCTGCTGG-3’

ACE_HRE#3_AS: 5’-CCCAACCGTGCCCTCT-3’

The following primers were used for PCR amplification of gene-lacking regions on chromosomes 5 and 6, which serve as inner reference controls in the calculation of relative enrichment levels:

mouse_Chr5_S: 5’-CCCGTCACTCAACCATTTCA-3’

mouse_Chr5_AS: 5’-CTTATCAATGGGGGCTCTGG-3’

mouse_Chr6_S: 5’-AGATATGGCTGGCTTTGTGC-3’

mouse_Chr6_AS: 5’-GAACTCGCTCAGGTTCTGC-3’


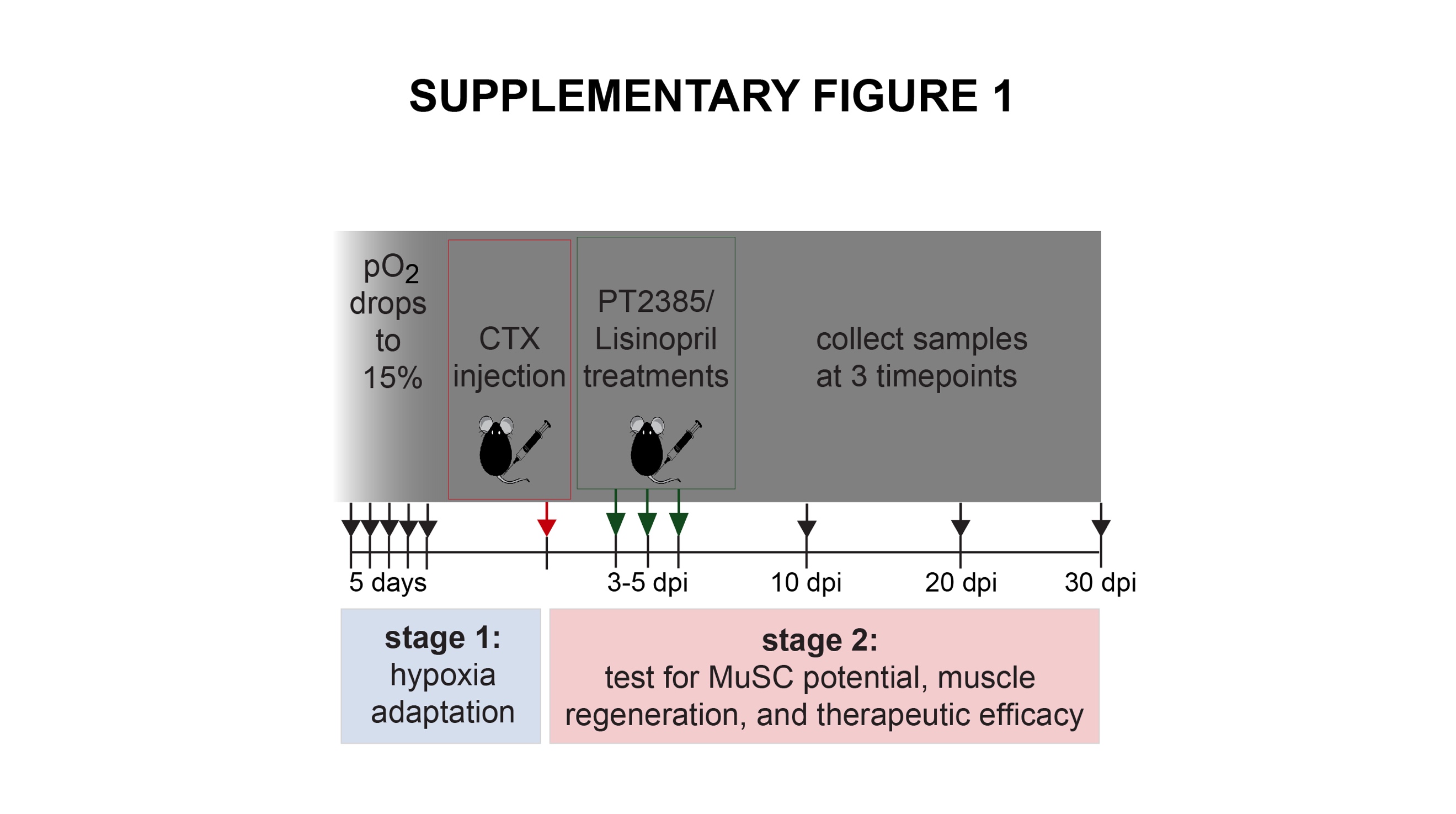


**Fig. S1 (associated with Fig. 1)**

Experimental design and timeline in this study.


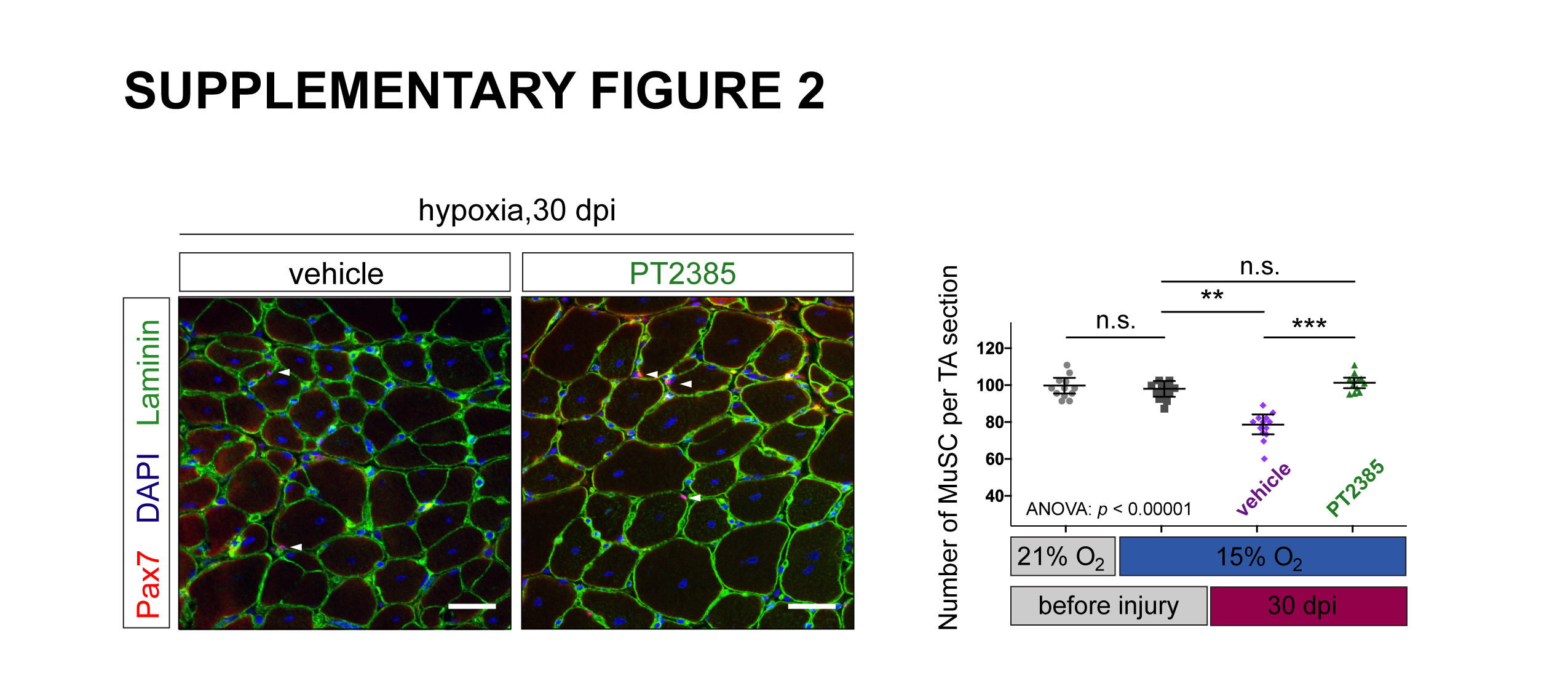


**Fig. S2 (associated with Fig. 4)**

**Left:** Representative IM images of Pax7 and Laminin B2 on TA muscle cross-sections from the vehicle or PT2385 treatment groups at 30 dpi. Arrowheads: Pax7^pos^ MuSC. Scale bar: 50 μm. **Right:** Quantification of Pax7^pos^ MuSC on TA muscle cross-sections in mice under nomoxia+CR (light grey) and hypoxia conditions (dark grey) at the end of the 2-week hypoxia adaptation stage (before injury) and from mice at 30 dpi after muscle regeneration under hypoxia but treated with either vehicle (purple) or PT2385 (green). (n=12 per group, 6 males and 6 females). **Statistical analysis legend**: in the right panel, one-way ANOVA was conducted to assess the equality of means among normoxia, normoxia+CR, and hypoxia groups treated with vehicle or PT2385. When *p*-value < 0.05 (rejection of the null hypothesis that all four groups have equal means), Post Hoc Tukey HSD tests were performed to determine significant differences between pairs of groups. The results of these Post Hoc tests have been denoted between the respective pairs of groups: ***: *p*-value < 0.001, **: *p*-value < 0.01, *: *p*-value < 0.05, n.s.: not significant. Error bars represent standard deviations (SD).

**SUPPLEMENTARY REFERENCES**

S1. Hawley JA, Lundby C, Cotter JD, and Burke LM. Maximizing Cellular Adaptation to Endurance Exercise in Skeletal Muscle. *Cell Metab.* 2018;27(5):962-76.

S2. Fry CS, Lee JD, Mula J, Kirby TJ, Jackson JR, Liu F, et al. Inducible depletion of satellite cells in adult, sedentary mice impairs muscle regenerative capacity without affecting sarcopenia. *Nat Med.* 2015;21(1):76-80.

S3. Keefe AC, Lawson JA, Flygare SD, Fox ZD, Colasanto MP, Mathew SJ, et al. Muscle stem cells contribute to myofibres in sedentary adult mice. *Nat Commun.* 2015;6:7087.

S4. Roman W, Pinheiro H, Pimentel MR, Segales J, Oliveira LM, Garcia-Dominguez E, et al. Muscle repair after physiological damage relies on nuclear migration for cellular reconstruction. *Science.* 2021;374(6565):355-9.

S5. Schmitt RE, Dasgupta A, Arneson-Wissink PC, Datta S, Ducharme AM, and Doles JD. Muscle stem cells contribute to long-term tissue repletion following surgical sepsis. *J Cachexia Sarcopenia Muscle.* 2023;14(3):1424-40.

S6. Sousa-Victor P, Gutarra S, Garcia-Prat L, Rodriguez-Ubreva J, Ortet L, Ruiz-Bonilla V, et al. Geriatric muscle stem cells switch reversible quiescence into senescence. *Nature.* 2014;506(7488):316-21.

S7. Zhou X, Wang JL, Lu J, Song Y, Kwak KS, Jiao Q, et al. Reversal of cancer cachexia and muscle wasting by ActRIIB antagonism leads to prolonged survival. *Cell.* 2010;142(4):531-43.

S8. Snijders T, Verdijk LB, and van Loon LJ. The impact of sarcopenia and exercise training on skeletal muscle satellite cells. *Ageing Res Rev.* 2009;8(4):328-38.

S9. He WA, Berardi E, Cardillo VM, Acharyya S, Aulino P, Thomas-Ahner J, et al. NF-kappaB-mediated Pax7 dysregulation in the muscle microenvironment promotes cancer cachexia. *J Clin Invest.* 2013;123(11):4821-35.

S10. Hauerslev S, Vissing J, and Krag TO. Muscle atrophy reversed by growth factor activation of satellite cells in a mouse muscle atrophy model. *PLoS One.* 2014;9(6):e100594.

S11. Keith B, Johnson RS, and Simon MC. HIF1alpha and HIF2alpha: sibling rivalry in hypoxic tumour growth and progression. *Nat Rev Cancer.* 2011;12(1):9-22.

S12. Koh MY, and Powis G. Passing the baton: the HIF switch. *Trends Biochem Sci.* 2012;37(9):364-72.

S13. Beaudry M, Hidalgo M, Launay T, Bello V, and Darribere T. Regulation of myogenesis by environmental hypoxia. *J Cell Sci.* 2016;129(15):2887-96.

S14. Majmundar AJ, Lee DS, Skuli N, Mesquita RC, Kim MN, Yodh AG, et al. HIF modulation of Wnt signaling regulates skeletal myogenesis in vivo. *Development.* 2015;142(14):2405-12.

S15. Yang X, Yang S, Wang C, and Kuang S. The hypoxia-inducible factors HIF1alpha and HIF2alpha are dispensable for embryonic muscle development but essential for postnatal muscle regeneration. *J Biol Chem.* 2017;292(14):5981-91.

S16. Zhu P, Hamlish NX, Thakkar AV, Steffeck AWT, Rendleman EJ, Khan NH, et al. BMAL1 drives muscle repair through control of hypoxic NAD(+) regeneration in satellite cells. *Genes Dev.* 2022;36(3-4):149-66.

S17. Wang X, Jia Y, Zhao J, Lesner NP, Menezes CJ, Shelton SD, et al. A mitofusin 2/HIF1alpha axis sets a maturation checkpoint in regenerating skeletal muscle. *J Clin Invest.* 2022;132(23).

S18. Mamchaoui K, Trollet C, Bigot A, Negroni E, Chaouch S, Wolff A, et al. Immortalized pathological human myoblasts: towards a universal tool for the study of neuromuscular disorders. *Skelet Muscle.* 2011;1:34.

S19. Yu T, Tang B, and Sun X. Development of Inhibitors Targeting Hypoxia-Inducible Factor 1 and 2 for Cancer Therapy. *Yonsei Med J.* 2017;58(3):489-96.

S20. Dubuisson N, Versele R, Planchon C, Selvais CM, Noel L, Abou-Samra M, et al. Histological Methods to Assess Skeletal Muscle Degeneration and Regeneration in Duchenne Muscular Dystrophy. *Int J Mol Sci.* 2022;23(24).

S21. Ordway GA, and Garry DJ. Myoglobin: an essential hemoprotein in striated muscle. *J Exp Biol.* 2004;207(Pt 20):3441-6.

S22. Ly CH, Lynch GS, and Ryall JG. A Metabolic Roadmap for Somatic Stem Cell Fate. *Cell Metab.* 2020;31(6):1052-67.

S23. Ohneda O, Nagano M, and Fujii-Kuriyama Y. Role of hypoxia-inducible factor-2alpha in endothelial development and hematopoiesis. *Methods Enzymol.* 2007;435:199-218.

S24. Christov C, Chretien F, Abou-Khalil R, Bassez G, Vallet G, Authier FJ, et al. Muscle satellite cells and endothelial cells: close neighbors and privileged partners. *Mol Biol Cell.* 2007;18(4):1397-409.

S25. Verma M, Asakura Y, Murakonda BSR, Pengo T, Latroche C, Chazaud B, et al. Muscle Satellite Cell Cross-Talk with a Vascular Niche Maintains Quiescence via VEGF and Notch Signaling. *Cell Stem Cell.* 2018;23(4):530-43 e9.

S26. Chazaud B. Inflammation and Skeletal Muscle Regeneration: Leave It to the Macrophages! *Trends Immunol.* 2020;41(6):481-92.

S27. Peng Y, Cui C, He Y, Ouzhuluobu, Zhang H, Yang D, et al. Down-Regulation of EPAS1 Transcription and Genetic Adaptation of Tibetans to High-Altitude Hypoxia. *Mol Biol Evol.* 2017;34(4):818-30.

S28. Chaouat A, Naeije R, and Weitzenblum E. Pulmonary hypertension in COPD. *Eur Respir J.* 2008;32(5):1371-85.

S29. Semprun-Prieto LC, Sukhanov S, Yoshida T, Rezk BM, Gonzalez-Villalobos RA, Vaughn C, et al. Angiotensin II induced catabolic effect and muscle atrophy are redox dependent. *Biochem Biophys Res Commun.* 2011;409(2):217-21.

S30. Yoshida T, Tabony AM, Galvez S, Mitch WE, Higashi Y, Sukhanov S, et al. Molecular mechanisms and signaling pathways of angiotensin II-induced muscle wasting: potential therapeutic targets for cardiac cachexia. *Int J Biochem Cell Biol.* 2013;45(10):2322-32.

S31. De Brandt J, Beijers R, Chiles J, Maddocks M, McDonald MN, Schols A, et al. Update on the Etiology, Assessment, and Management of COPD Cachexia: Considerations for the Clinician. *Int J Chron Obstruct Pulmon Dis.* 2022;17:2957-76.

S32. Nangaku M, and Fujita T. Activation of the renin-angiotensin system and chronic hypoxia of the kidney. *Hypertens Res.* 2008;31(2):175-84.

S33. Sukhanov S, Semprun-Prieto L, Yoshida T, Michael Tabony A, Higashi Y, Galvez S, et al. Angiotensin II, oxidative stress and skeletal muscle wasting. *Am J Med Sci.* 2011;342(2):143-7.

S34. Song YH, Li Y, Du J, Mitch WE, Rosenthal N, and Delafontaine P. Muscle-specific expression of IGF-1 blocks angiotensin II-induced skeletal muscle wasting. *J Clin Invest.* 2005;115(2):451-8.

S35. Zhang L, Du J, Hu Z, Han G, Delafontaine P, Garcia G, et al. IL-6 and serum amyloid A synergy mediates angiotensin II-induced muscle wasting. *J Am Soc Nephrol.* 2009;20(3):604-12.
